# Atlantic Origin of the Arctic Biota? Evidence from Phylogenetic and Biogeographical Analysis of the Cheilostome Bryozoan Genus *Pseudoflustra*

**DOI:** 10.1371/journal.pone.0059152

**Published:** 2013-03-25

**Authors:** Piotr Kuklinski, Paul D. Taylor, Nina V. Denisenko, Björn Berning

**Affiliations:** 1 Institute of Oceanology, Polish Academy of Sciences, Sopot, Poland; 2 Natural History Museum, London, United Kingdom; 3 Zoological Institute, Russian Academy of Sciences, St. Petersburg, Russia; 4 Geoscientific Collections, Upper Austrian State Museum, Leonding, Austria; Consiglio Nazionale delle Ricerche (CNR), Italy

## Abstract

The intricate geological evolution of the Arctic Ocean is paralleled by complexities in the biogeographical and phylogenetical histories of the Arctic biota, including bryozoans. Here we present revised taxonomic descriptions for all known species of the bryozoan genus *Pseudoflustra*, and use the present-day distributions and phylogenetic relationships between these species to infer the historical biogeography of the genus. Nine species belonging to the genus *Pseudoflustra* are recognized in the Arctic and North Atlantic. One new species, previously identified as *Ichthyaria aviculata*, is described as *Pseudoflustra radeki* sp. nov. Another species, previously assigned to *Smittoidea* as *S. perrieri*, is transferred to *Pseudoflustra*. Biogeographical analysis of *Pseudoflustra* reveals that species distributions mostly match current patterns pertaining in the North Atlantic and Arctic Ocean. Distributions were probably shaped by recent geological history as present-day current directions in the Arctic Ocean are believed to have been similar for at least the last 120 000 years. Phylogenetic analysis of *Pseudoflustra* places the five Arctic-North Atlantic species in a clade crownward of a paraphyletic grouping of North Atlantic species. Given that the Arctic Ocean was fully glaciated until 18 000 years, the most likely explanation for this phylogeographical pattern is that species of *Pseudoflustra* colonized the Arctic relatively recently from North Atlantic sources. However, a fuller understanding of the origin of *Pseudoflustra* in the Arctic will require molecular and fossil data, neither of which are currently available.

## Introduction

Animal and plant species making up the marine biota currently inhabiting the Arctic Ocean have undergone varied evolutionary histories. It is believed that the Arctic shelf benthic fauna and flora is composed predominantly of North Atlantic emigrants, and that little species exchange has occurred with the North Pacific [1]. However, molecular studies of the red alga *Phycodrys rubens*, which is currently distributed throughout the Arctic and North Atlantic, show that it originated in the Pacific and colonized the North Atlantic after the opening of the Bering Strait about 3–3.5 Ma [2]. There are also a number of similar examples among Arctic bivalves and gastropods [3], [4], while the fossil record points to repeated dispersal events from the Pacific Ocean via the Bering Strait into the Arctic and the North Atlantic [5].

Complexities in the biogeographical histories of the Arctic biota parallel intricacies in geological evolution of the Arctic Ocean basin [6], [7], [8]. From at least the latest Cretaceous (80–65 Ma) the Arctic basin had been cut-off from the Pacific Ocean [6] but at the end of the Miocene (5.32 Ma) the Bering Strait opened, re-establishing the connection [7]. During Eocene times (ca 40 Ma), the Arctic Ocean opened into the North Atlantic, allowing exchange of biotas between these two regions. Subduction of the Iceland Ridge below sea level in the mid Miocene (15–10 Ma) allowed deep-water exchange between the Arctic and North Atlantic [6]. This probably led to the development of a distinct boreal marine province in the Arctic, and to the evolution of a cool, boreal biota of Atlantic character. More recently, during the last major glaciation of the Arctic, which reached its zenith around 18 000 years ago and ended some 6000 years ago, vast areas of the Arctic continental shelf were covered by ice sheets [1], [3], [8]. These ice sheets extended over North America, Greenland, Iceland, Scandinavia, the Barents Sea and the Kara Sea; only parts of the continental shelves of the Chukchi, Beaufort and East Siberian seas remained largely unglaciated, despite being almost entirely emergent [1], [8]. During this period the Arctic shelf fauna was almost eradicated. Reinvasion of the Arctic is thought to have taken place after the ice began to retreat about 14 000 years ago. Colonization was accomplished by the few survivors that had been able to retreat into the North Atlantic or North Pacific, or had taken refuge in unglaciated shelf areas of the East Siberian Sea, the Beaufort Sea or deeper bathyal parts of the Arctic Ocean [1], [3].

Among the commonest inhabitants of shallow marine environments in the Arctic Ocean today are bryozoans. However, incomplete knowledge of bryozoan taxonomy and distributions has hampered the exploitation of their potential in understanding the historical biogeography of the Arctic. Many recent phylogeographical analyses of the Arctic biota have focused on planktonic taxa or benthic taxa with planktonic larvae that can be widely dispersed by oceanic currents [9], [10], [11]. In contrast, the majority of bryozoan species are sessile and benthic as adults, becoming dispersed via lecitotrophic larvae that stay in the water column for a matter of a few hours only [12]. This life history should retard the speed of colonization of new areas, leading to the expectation that bryozoan species will be less widespread than planktonic taxa or benthic taxa with planktonic larvae.

The current paper uses a taxonomic revision of the cheilostome bryozoan genus *Pseudoflustra* to infer its phylogeography. This genus was believed to be indigenous to the Arctic and consist of only five species [13] until d’Hondt [14] transferred *Ichthyaria aviculata* Calvet, 1906, a temperate North Atlantic species, into *Pseudoflustra*. Subsequently, Hayward [15] described two new species (*P. minima* and *P. virgula*) from extensive samples collected during the BIOFAR programme around the Faroe Islands, also in the North Atlantic. The present paper provides revised taxonomic descriptions of all known and one new species of *Pseudoflustra*, and uses the present-day distributions and phylogenetic relationships between its constituent species to infer the historical biogeography of the genus.

## Materials and Methods

The studied material is lodged in the following repositories: Natural History Museum, London (NHM); University of Manchester Museum (MM); Museum Nationale d’Histoire Naturelle, Paris (MNHN); Naturhistorisches Museum, Vienna (NHMV); Canadian Museum of Nature, Ottawa (CMN); Zoological Museum, Copenhagen (ZM) and the Zoological Institute of the Russian Academy of Sciences, St Petersburg (ZI). Permissions from all museums were obtained to access and study their collections. In some cases (ZM) material was loaned while in all other cases investigation were done on site.

Both historical and new collections were employed for this study. Samples used in this study were preserved in ethanol or dry. Unregistered material was collected around Faroe Islands during the BIOFAR project. No specific permits were required for any of the field collections and the sampling sites were neither privately owned nor protected. None of the samples were of endangered or protected species.

Scanning electron microscopy (SEM) was undertaken using a low-vacuum instrument (LEO 1455-VP) for imaging uncoated specimens with back-scattered electrons. Prior to scanning, some samples were bleached in a 10% solution of sodium hypochlorite in order to remove all organic material obscuring the important taxonomic features of the skeleton. Measurements of zooid size were made on SEM images using the software ImageJ. There are strong seasonal differences in food supply in the Arctic owing to the fact that the polar night lasts for almost three months each year. Therefore, large differences in these measurements can be observed. To minimize seasonal bias, for each species 60 measurements were made from at least three specimens from different localities. Measurements are summarized in Table 1.

**Table 1:** Measurements of zooidal characteristics (mean ± SD [mm], number of measurements in parentheses).

	*P. solida*	*P. hincksi*	*P. anderssoni*	*P. sinuosa*	*P. birulai*	*P. minima*	*P. virgula*	*P. radeki*	*P. perrieri*
Autozooid length	1.110	0.951	0.879	1.663	1.263	0.858	0.847	0.738	0.783
	±0.133 (60)	±0.159 (60)	±0.146 (60)	±0.261 (60)	±0.208 (60)	±0.058 (21)	±0.056 (20)	±0.145 (10)	±0.063 (30)
Autozooid width	0.312	0.230	0.266	0.434	0.288	0.239	0.437	0.267	0.384
	±0.031 (60)	±0.030 (60)	±0.030 (60)	±0.075 (60)	±0.030 (60)	±0.019 (11)	±0.065 (20)	±0.061 (11)	±0.033 (30)
Orifice length	137.000	0.121	0.131	0.172	0.117	0.109	0.146	0.120	0.136
	±0.012 (60)	±0.012 (60)	±0.010 (60)	±0.014 (60)	±0.013 (60)	±0.012 (20)	±0.014 (20)	±0.014 (12)	±0.016 (30)
Orifice width	0.181	0.122	0.148	0.230	0.170	0.108	0.144	0.125	0.129
	±0.012 (60)	±0.010 (60)	±0.010 (60)	±0.012 (60)	±0.013 (60)	±0.012 (13)	±0.019 (20)	±0.008 (9)	±0.010 (19)
Avicularium length	0.244	0.117	0.161	0.285	0.100	0.148	0.182	0.101	0.166
	±0.044 (60)	±0.008 (60)	±0.023 (60)	±0.061 (60)	±0.013 (31)	±0.011 (12)	±0.020 (20)	±0.010 (13)	±0.013 (30)
Avicularium width	0.124	0.073	0.109	0.156	0.114	0.087	0.140	0.074	0.122
	±0.008 (60)	±0.006 (60)	±0.009 (60)	±0.031 (60)	±0.014 (60)	±0.008 (7)	±0.015 (20)	±0.011 (11)	±0.011 (30)
Distance betweenorifice andavicularium	0.058	0.036	0.035	0.095	–	0.075	0.041	0.010	0.017
	±0.008 (60)	±0.005 (60)	±0.005 (60)	±0.014	–	±0.009 (19)	±0.010 (30)	±0.004 (12)	±0.005 (30)
Ovicell length	0.302	0.277	0.281	0.475	0.312	0.231	0.286	0.320	0.354
	±0.014 (60)	±0.023 (60)	±0.029 (60)	±0.087 (60)	±0.025 (60)	(1)	±0.025 (18)	±0.003 (2)	±0.001 (2)
Ovicell width	0.327	0.281	0.288	0.495	0.368	0.267	0.359	0.344	0.380
	±0.021 (60)	±0.021 (60)	±0.028 (60)	±0.092 (60)	±0.025 (60)	(1)	±0.025 (17)	±0.025 (2)	±0.025 (2)
Ovicell pores	16.47	7.55	7.30	20.40	2.75	24 (1)	8.33	27.00	4.50
	±3.41 (20)	±1.50 (20)	±2.67 (20)	±2.11 (20)	±0.85 (20)	(1)	±1.50 (6)	±1.41 (2)	±0.70 (2)

Cladistic analysis was undertaken using PAUP 4.0®.

### Nomenclatural Acts

The electronic edition of this article conforms to the requirements of the amended International Code of Zoological Nomenclature, and hence the new names contained herein are available under that Code from the electronic edition of this article. This published work and the nomenclatural acts it contains have been registered in ZooBank, the online registration system for the ICZN. The ZooBank LSIDs (Life Science Identifiers) can be resolved and the associated information viewed through any standard web browser by appending the LSID to the prefix “http://zoobank.org/”. The LSID for this publication is: urn:lsid:zoobank.org:pub: 6838A698-5EC4-4BFF-827D-5918B8AD916C. The electronic edition of this work was published in a journal with an ISSN, and has been archived and is available from the following digital repositories: PubMed Central, LOCKSS.

## Results

### Systematics

Order **CHEILOSTOMATA** Busk, 1852

Suborder **NEOCHEILOSTOMINA** d'Hondt, 1985

Superfamily **SMITTINOIDEA** Levinsen, 1909

Family **SMITTINIDAE** Levinsen, 1909

Genus ***Pseudoflustra*** Bidenkap, 1897

### Diagnosis (revised)

**Figure 1:**
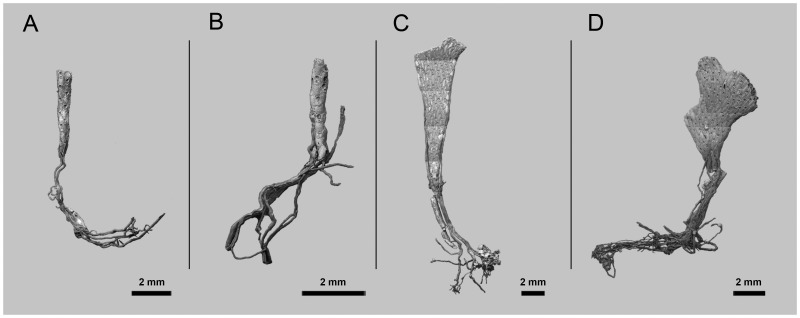
Examples of *Pseudoflustra* colonies at different astogenetic stages showing the rootlets enabling attachment to the substrate. A. *P. anderssoni* (ZI RAS 27/1764); B. *P. anderssoni* (ZI RAS 27/1764); C. *P. hincksi* (ZI RAS 27/1764); D. *P. hincksi* (ZI RAS 3).

Colony erect, bifoliate or vincularian, branches or fronds often widening distally, sometimes bifurcating; cuticular rootlets developed proximally, thick, initially adherent to colony surface before entering substrate in bundles (Figure 1). Autozooids arranged in straight longitudinal rows, either around entire circumference of branches or more often restricted to frontal surface of flat bilaminar branches; oblong, often narrowing proximally, separated by shallow grooves; frontal shield umbonuloid, smooth or sometimes finely granular, imperforate except for areolar pores along lateral edges, variously shaped; primary orifice oval with condyles at proximolateral corners, with or without a sinus or a central denticle (lyrula); oral spines absent; secondary orifice oval, cormidial, distal and sometimes lateral parts formed by calcification of adjacent zooids. Ovicell hyperstomial, round, globular, endo- and ectooecium calcified, exposed ectooecium smooth, pseudoporous, the pseudopores varying in shape, size and distribution, surface often overgrown from lateral and distal sides by calcification from surrounding zooids. Avicularia adventitious, suboral, triangular or oval, directed proximally, rarely absent; additional proximolateral avicularia occasionally developed in some species.

Type species: *Flustra solida* Stimpson, 1854.

### Remarks

The type species of *Pseudoflustra*, originally placed by Stimpson [16] in the anascan genus *Flustra*, was transferred by Sars [17] to the unrecognizable ascophoran genus *Eschara* Linneaus, 1758. Soon afterwards it was reassigned by Smitt [18] to *Escharella* Gray, 1848, and subsequently by Verrill [19] to *Flustramorpha* Gray, 1872. Hincks [20], [21] reverted to *Flustra* while stating that *F. solida* only superficially resembled this genus and that there was no real affinity. It was left to Bidenkap [22] to introduce the new genus *Pseudoflustra*.

*Pseudoflustra* has an umbonulomorph frontal shield and has generally been placed in the family Smittinidae Levinsen, 1909. As early as 1892, Hincks [21] recognized the resemblance of *Pseudoflustra* to *Smittina* [*Smittoidea*] *reticulata*, pointing out that they differed only in the presence of denticles and marginal spines in the orifice of the latter.

Genera of Smittinidae have been classified in Lepraliomorpha, a group of ascophoran cheilostomes with lepralioid frontal shields. However, recent molecular studies indicate that lepralioid frontal shields have evolved more than once in several different cheilostome clades. Waeschenbach et al. [23], for example, showed sister group relationships between various umbonuloid and lepralioid species such as the umbonuloid *Umbonula litoralis* and the lepralioid *Pentapora foliacea*. Smittinidae probably represent another example of mixed umbonuloid and lepralioid frontal shields as at least the species of *Pseudoflustra* are umbonuloid, which is evidenced by the presence of a ring scar.

Cheilostome bryozoan taxonomy is based almost entirely on the skeletal morphology of the zooids. Of particular taxonomic importance in ascophoran cheilostomes is the shape of the primary orifice, including whether it has a sinus and/or a lyrula or central denticle [24]. Yet, as Kluge [25] pointed out, such characteristics of the orifice differ between species of *Pseudoflustra*. In some species the orifice has a rather straight proximal margin (*P. solida*, *P. virgula*, *P. radeki*), in a few there is a well-defined sinus (*P. hincksi*, *P. anderssoni*), while in others a lyrula or a proximal denticle is developed (*P. sinuosa*, *P. birulai*, *P. perierri*). Normally such differences would be taken to justify a placement in different genera or even families. For example, some species (e.g. *P. anderssoni*) have an orifice resembling that of *Schizoporella* (Family Schizoporellidae Jullien, 1883), whereas in others (e.g. *P. sinuosa*) the orifice is similar to that of *Smittina* (Family Smittinidae Levinsen, 1909). In spite of these variations, the unity of *Pseudoflustra* is supported by the occurrence of shared characters likely to be apomorphic for the genus. The nine species described below all have erect colonies with very distinct rootlets originating from the cuticular walls of proximal autozooids (Figure 1). While cuticular rootlets are known in a wide range of ascophoran cheilostomes (e.g. *Cellarinella* Waters, 1904, *Adeona* Lamouroux, 1812, *Margaretta* Gray, 1843), features of the zooidal skeleton in *Pseudoflustra* are distinct from those of other rooted ascophorans. Additional features shared between all nine species of *Pseudoflustra* are the presence of condyles in the proximolateral corners of the primary orifice, the cormidial secondary orifice, the oval or triangular suboral avicularium, and the globular hyperstomial ovicell with a variably pseudoporous ectooecium that is often overgrown by calcification from the surrounding zooids.

Several cheilostome genera known from the Atlantic and Pacific oceans resemble *Pseudoflustra*. Among these, *Smittoidea* Osburn, 1952, which occurs in both the North Atlantic and Pacific, shows the greatest similarities and may be the most closely related living genus. All species of *Smittoidea* possess a lyrula and a frontal shield without pseudopores. Tellingly, at least one species of *Pseudoflustra*, *P. perrieri* (Julien, 1883), has been referred to *Smittoidea* in relatively recent times (1978) [26]. However, unlike *Pseudoflustra*, species of *Smittoidea* are typically encrusting and some have oral spines (e.g. *S. prolifica* Osburn, 1952), which are lacking in *Pseudoflustra*.

### Key to Species of the Genus *Pseudoflustra*

1. Colony erect, vinculariiform with autozooidal orifices opening only on the frontal surface ***P. radeki***
  - Colony erect, vinculariiform with autozooidal orifices distributed circumferentially 2
  - Colony erect, flat, bifoliate 4
2. Avicularia suboral, oval ***P. minima***
  - Avicularia suboral, longer than wide, directed proximally, with rostrum triangular. 3
3. Denticle on the proximal margin of the primary orifice ***P. perrieri***
  - Proximal margin of the primary orifice straight ***P. virgula***
4. Primary orifice with denticle on proximal margin 5
  - Primary orifice lacking a denticle 6
5. Denticle broad, sometimes expanding outwardly ***P. sinuosa***
  - Denticle small and sharp ***P. birulai***
6. Primary orifice with broad sinus at the proximal margin 7
  - Primary orifice without a sinus; proximal margin relatively straight ***P. solida***
7. Cryptocyst present between orifice and suboral avicularium ***P. anderssoni***
  - Cryptocyst lacking between orifice and suboral avicularium ***P. hincksi***

**Figure 2:**
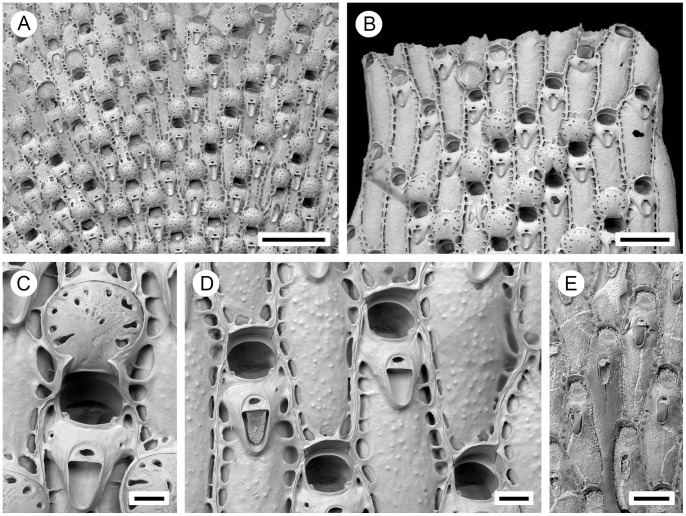
*Pseudoflustra solida* (Stimpson, 1854). A. colony showing autozooids and ovicellate zooids, bleached; B. distal colony edge with autozooids and ovicellate zooids, bleached; C. ovicellate zooid with avicularium, bleached; D. autozooids with avicularia, bleached; E. characteristic chitinized tube originated from cuticle covering autozooidal frontal shield and stretching along the surface of the colony before detaching to become a rootlet, unbleached. A, ZI RAS 70/2942; B, C, D, ZI RAS 66; E, MM 4163. Scale bars: 1 mm (A), 500 µm (B), 300 µm (E), 100 µm (C, D).

***Pseudoflustra solida*** (Stimpson, 1854)

(Figures 2A–E, Table 1)

*Flustra solida* Stimpson, 1854: 19, fig. 12 [16]; Hincks, 1880: 282, pl. 15, figs 2, 3 [20]; ?1892: 149, pl. 8, figs. 1b–c [21].

**Figure 3:**
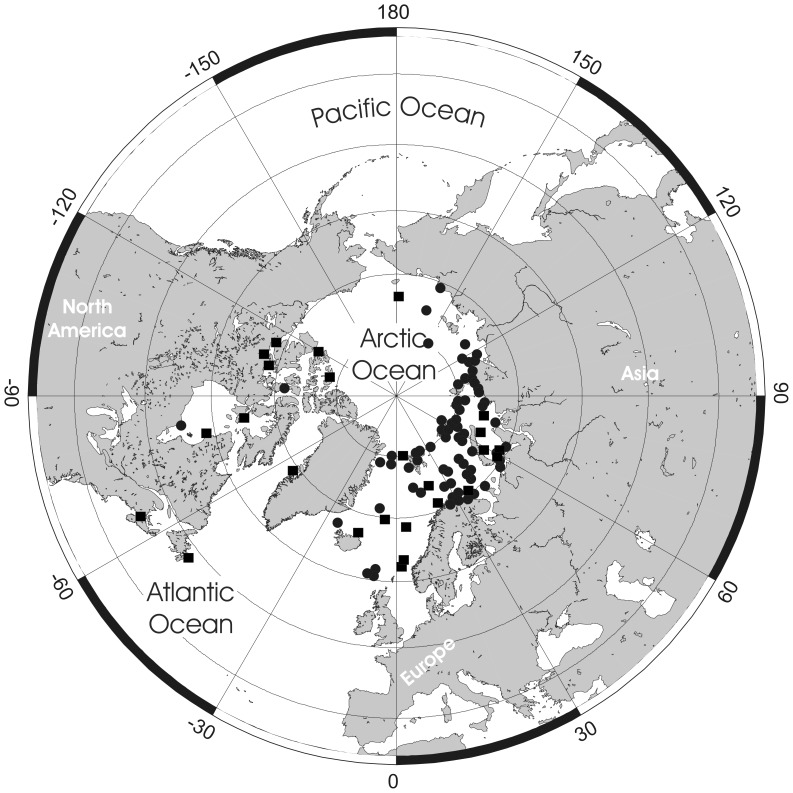
Distributional records of *Pseudoflustra solida* gathered from the literature and museum collections. (circles – record confirmed either by scanning electron or light microscopy, squares – unconfirmed record taken from literature). Note that some symbols represent more than one record as the resolution of the map is insufficient to depict all records.

*Escharella palmata* Smitt, 1868: 10, pl. 24, figs 42–46 [27].

*Eschara solida* (Stimpson) – Vigelius, 1882: 15, figs 2, 3a–b [28].

**Figure 4:**
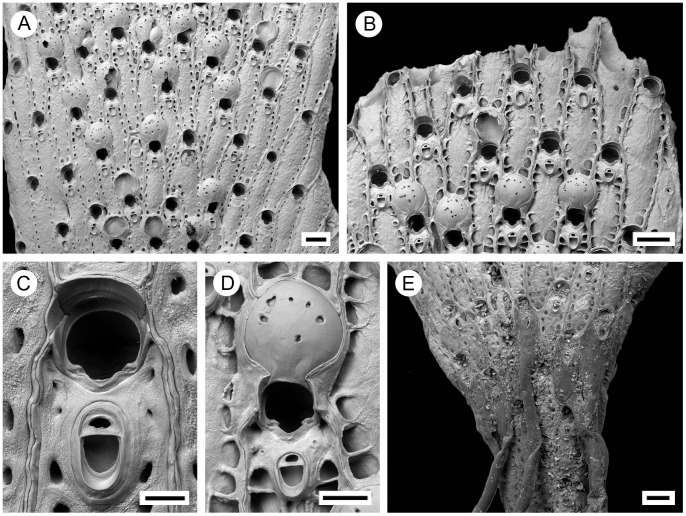
*Pseudoflustra hincksi* Kluge, 1915. A. colony showing autozooids and ovicellate zooids, bleached; B. distal colony edge with autozooids and ovicellate zooids, bleached; C. orifice of autozooid with avicularium, bleached; D. ovicellate zooid with suboral avicularium, bleached; E. characteristic chitinized cuticular rhizoids growing proximally as inflations of frontal shield cuticles of autozooids, unbleached. A, ZI RAS 12; B, D, ZI RAS 33/5528; C, E, ZI RAS 3. Scale bars: 200 µm (A, B, E), 100 µm (D), 50 µm (C).

*Pseudoflustra solida* (Stimpson) – ?Marcus, 1919: 271 [29]; Kluge, 1962: 441, fig. 292 [30]; Kluge, 1975: 535, fig. 292 [25]; Powell, 1968: 2309, pl. 12, fig. c [13]; Gontar and Denisenko, 1989, pl. 14-IV, fig. 4 [31]; NOT: Ryland and Hayward, 1991: 41, fig. 64 [32].

### Material Examined

See Appendix S1.

### Description

Colony erect, flat, bifoliate, branches widening towards the distal end, sometimes bifurcating, basally rooted with cuticular rhizoids growing proximally as inflations of frontal cuticles of autozooids, the rhizoids initially adherent to colony surface before clustering into thick bundles that extend into the substrate (Figures 1, 2A). Ancestrula unknown.

Autozooids arranged in straight longitudinal rows numbering up to 26, slender, oblong-rectangular, often narrowing proximally, separated by shallow grooves, large (Figures 2A–B). Frontal shield smooth or sometimes finely granular, bordered laterally by conspicuous oval or elongated-oval areolar pores often with thickened edges opening towards centre of shield (Figure 2D). Primary orifice oval, wider than long, distal border a smooth curved shelf; triangular condyle at each proximolateral corner; secondary orifice oval, cormidial, distal and sometimes lateral parts formed from calcification of neighbouring zooids (Figure 2C).

Ovicell hyperstomial, globular, lateral and distal border encapsulated and overgrown by calcification of neighbouring zooids, slightly broader than long, large; ectooecium pseudoporous with 12–23 (mean 16, n = 20) pseudopores of irregular size and shape scattered over entire surface, largest near periphery (Figure 2C).

Avicularia adventitious, suboral, large, longer than wide, directed proximally, rarely absent; rostrum barely narrowing distally with round tip, distal shelf absent; mandible elongated, ligulate; crossbar calcified, broad, the straight distal edge without columella; avicularian chamber inflated, occupying space between proximal edge of orifice and rostrum and spreading outwards to lateral margins of the zooid, sometimes with pores on lateral sides of the chamber (Figures 2C–D).

### Remarks

Attempts to locate the collection used by Stimpson [16] when introducing this species have been unsuccessful, despite enquiries to various museums in the USA (Washington, Harvard, Yale and Chicago). Most probably the type material was lost during the fire in 1866 of the Chicago Academy of Sciences where a large part of the Stimpson collection was formerly housed [33]. Stimpson’s description, together with his drawing of this species, is relatively undiagnostic. However, the type locality (Bay of Fundy, off the northern point of Duck Island) is within the geographical range of the species as interpreted by subsequent authors and there are no compelling reasons to revise the concept of the species or to select a neotype given that the identity of the species is not in doubt.

*Pseudoflustra solida* has several characters that make it relatively easy to distinguish from other species of *Pseudoflustra*. Firstly, it is one of only three species to have a relatively straight proximal edge to the primary orifice. The other two species – *P. virgula* and *P. radeki* – are temperate Atlantic species with vincularian colonies that are much smaller colonies than the bifoliate colonies of *P. solida*. Moreover, branches of *P. virgula* and *P. radeki* are composed of no more than seven rows of autozooids (*P. radeki* has only two rows of autozooids), whereas in *P. solida* branches can consist of at least 26 autozooidal rows per side. *P. virgula* has triangular, drop-like avicularia, while both *P. solida* and *P. radeki* have a rounded avicularium. In addition, *P.radeki* is the only species within the genus in which the autozooids are confined to one side (frontal) of the branch.

### Distribution

*Pseudoflustra solida* is the commonest Arctic species of *Pseudoflustra*. It has been recorded from depths between 20 and 1160 m, and from various regions in the Arctic Ocean between 109°W and 170°E (Figure 3). The lowest latitude from which it has been recorded is 48° N in the northwest Atlantic. A record of *P. solida* as a fossil in the Neogene of northern Japan [34] is most likely a misidentification as the presence of an orificial sinus and zooidal dimensions do not match true *P. solida*.

***Pseudoflustra hincksi*** Kluge, 1915

(Figures 4A–E, Table 1)

*Pseudoflustra hincksi* Kluge, 1915: 383 [35]; Kluge, 1946: 199, pl. 2, fig. 4 [36]; Kluge, 1962: 443, fig. 293 [30]; Kluge, 1975: 536, fig. 293 [25]; d’Hondt and Mascarell, 2004: 283 [37].

*Flustra solida* (Stimpson) – Hincks, 1892: 149, pl. 8, figs. 1, 1a [21].

### Type Material

Lectotype (here designated): ZI 6, Kola Bay, stn 22, *A. Pervozvanniy*, dredge, 12.06.1893, determined by G.A. Kluge, collected by N.M. Knipowich. This specimen from the Kluge Collection is here designated as the lectotype based on the match between the locality information and that given in the original description [35].

### Other Material Examined

See Appendix S1.

### Description

Colony erect, initially vincularian, becoming bifoliate in later astogeny with flattened branches broadening distally and sometimes bifurcating; basally rooted using cuticular rhizoids growing proximally as inflations of frontal cuticles of autozooids, initially adherent to colony surface before clustering into thick bundles that enter the substrate (Figures 1, 4E). Ancestrula unknown.

Autozooids arranged in straight rows, up to 19 on each side of branch, slender, oblong-rectangular, often narrowing proximally, separated by shallow grooves, large. Frontal shield smooth or sometimes finely granular; conspicuous areolae present along lateral margins, sometimes appearing to occupy entire frontal shield, often with thickened edges opening towards centre of shield, areolar pores oval or elongated-oval, minute to very large in size (Figures 4A, B). Primary orifice oval, on average as long as wide, sinus broad, distally with a smooth curved shelf, condyles at each proximolateral corner; secondary orifice oval, cormidial with distal and sometimes part of lateral perimeter constituted by calcification of neighbouring autozooids, slightly longer than wide, a weakly developed peristome sometimes developed laterally and proximally, concave proximal margin with shallow U-shaped pseudosinus (Figures 4C, D). Ovicell hyperstomial, round, globular, lateral and distal border encapsulated and overgrown by calcification of surrounding zooids, almost as long as wide; 6 to 12 (mean 8, n = 20) pseudopores of variable size and shape scattered irregularly over entire surface (Figures 4D).

Avicularia adventitious, suboral, large, longer than wide, directed proximally; rostrum oval with rounded tip, distal calcified shelf relatively narrow; mandible elongated, ligulate; crossbar calcified, broad; space between orifice and avicularia occupied by avicularian chamber which spreads to lateral margins of autozooid, sometimes porous laterally; in older zooids avicularian chamber becomes less visible (Figures 4C, D). Avicularia rarely absent.

### Remarks

Apart from *P. anderssoni*, this is the only species of *Pseudoflustra* to have a sinus in the primary orifice. However, *P. anderssoni* differs from *P. hincksi* in possessing an area of cryptocyst between the orifice and the suboral avicularium, as well as often developing lappets laterally around the secondary orifice and stretching down to the suboral avicularium. Additionally, the palate occupies a larger proportion of the avicularium in *P. anderssoni* than in *P. hincksi*.

### Distribution

**Figure 5:**
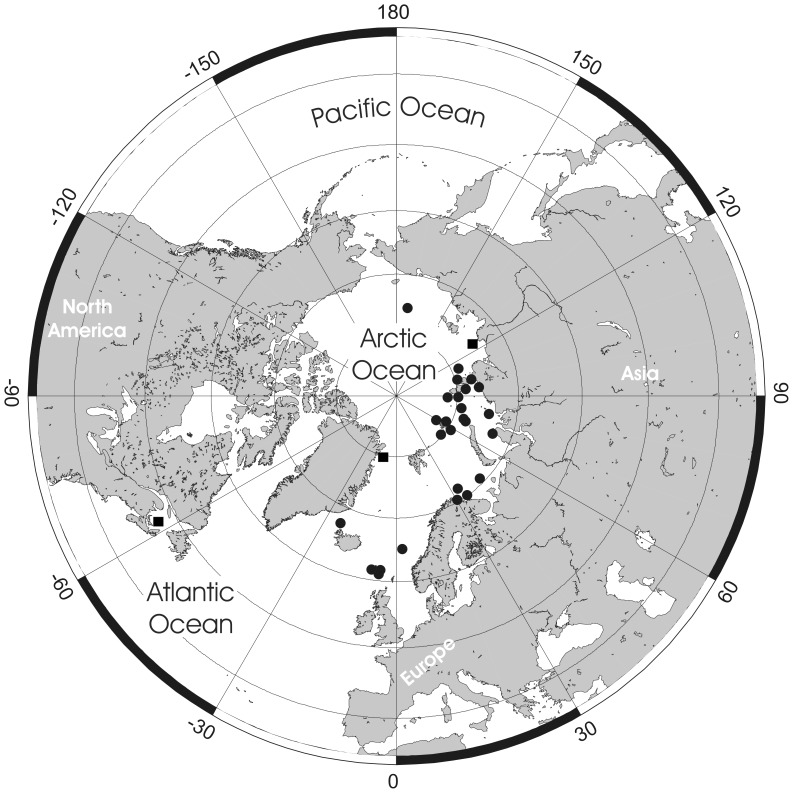
Distributional records of *Pseudoflustra hincksi* gathered from the literature and museum collections. (circles – record confirmed either by scanning electron or light microscopy, squares – unconfirmed record taken from literature). Note that some symbols represent more than one record as the resolution of the map is insufficient to depict all records.

The confirmed records of this Arctic species are from depths between 5 and 1075 m, and at longitudes between ∼28°W and 173°E, including localities in Greenland, the Barents, Kara, Laptev and East Siberian seas and the Gulf of St Lawrence [25]. The lowest latitude at which *P. hincksi* occurs is ∼47°N, ∼64°W (St. Lawrence, Bear Head, Anticosti) [21] (Figure 5), although this record is unconfirmed.

**Figure 6:**
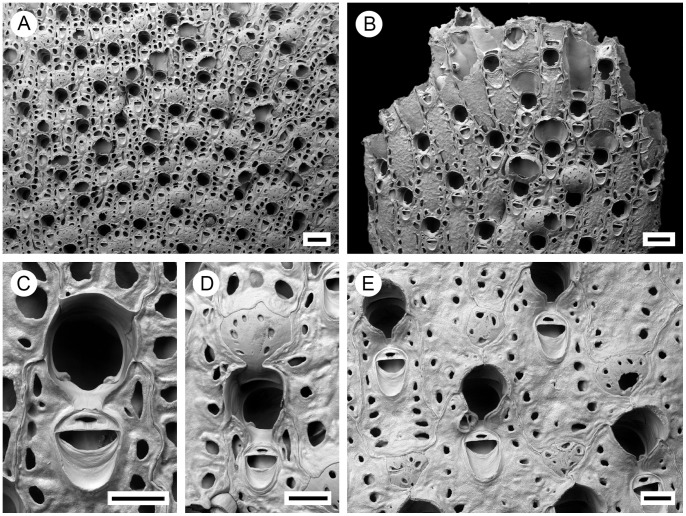
*Pseudoflustra anderssoni* Kluge, 1946. A. colony showing autozooids and ovicellate zooids, bleached; B. distal colony edge with autozooids and ovicellate zooids, bleached; C. orifice of autozooid with avicularium, bleached; D. ovicellate zooid with suboral avicularium, bleached; E. mid branch part of a colony with ovicellate zooids showing characteristic rare avicularium placed proximolaterally at the edge of the orifice, bleached. A, D, ZI RAS 2; B, C, ZI RAS 10; E, NHM 2012.3.7.2. Scale bars: 200 µm (A, B), 100 µm (C, D, E).

***Pseudoflustra anderssoni*** Kluge, 1946

(Figures 6A–E, Table 1)

*Pseudoflustra anderssoni* Kluge, 1946: 198, pl. 2, fig. 5 [36]; Kluge 1962: 444, fig. 294 [30]; Kluge 1975: 538, fig. 294 [25].

### Type Material

Lectotype (here designated): ZI 13/2456, Arctic Ocean, stn 93, *Sadko*, trawl, 12.04.1938, determined by G.A. Kluge, collected by G.P. Gorbunov. This specimen is here designated as the lectotype based on the match between the locality information and that given in the original description [36].

### Other Material Examined

See Appendix S1.

### Description

Colony erect, initially vincularian, becoming flat bifoliate in later astogeny with branches widening distally, sometimes bifurcating; basally rooted from cuticular rhizoids originating as tube-like prolongations of autozooidal frontal cuticles, growing proximally, initially adherent to colony surface before clustering into thick bundles rooting into soft substrates (Figure 1). Ancestrula unknown.

Autozooids arranged in straight longitudinal rows numbering up to 21 on each side of branch, slender, oblong-rectangular, often narrowing towards the proximal end, separated by shallow grooves. Frontal shield smooth or rarely finely granular, conspicuous areolar pores present along lateral margins, oval or elongated-oval, minute to large in size, sometimes appearing to occupy entire frontal shield, often with thickened edges, opening towards centre of shield (Figures 6A, B). Primary orifice round, slightly wider than long, with a shallow sinus proximally, condyles at proximolateral corners, smooth suboral shelf between orifice and avicularium; secondary orifice oval, cormidial, peristome distally and partly laterally constructed by calcification of neighbouring zooids, pseudosinus present proximally, lateral lobes of peristome often enclosing suboral avicularium (Figures 6C–D). Ovicell hyperstomial, round, globular, slightly broader than long, large, lateral and distal border overgrown at the edges by calcification of surrounding zooids, overgrowths from left and right sides sometimes fusing to form bridge across proximal part of ovicell, pseudopores numbering 4 to 12 (mean 7, n = 20), variable in size and shape, scattered over entire surface (Figures 6D–E).

Avicularia adventitious, rarely absent, suboral, large, longer than wide, directed proximally; rostrum oval with rounded tip, distal calcified shelf extensive; mandible elongate, ligulate; crossbar calcified, broad (Figures 6C–E).

### Remarks

*Pseudoflustra anderssoni* is closely related to *P. hincksi*. Differences are outlined in the remarks for *P. hincksi*.

### Distribution

**Figure 7:**
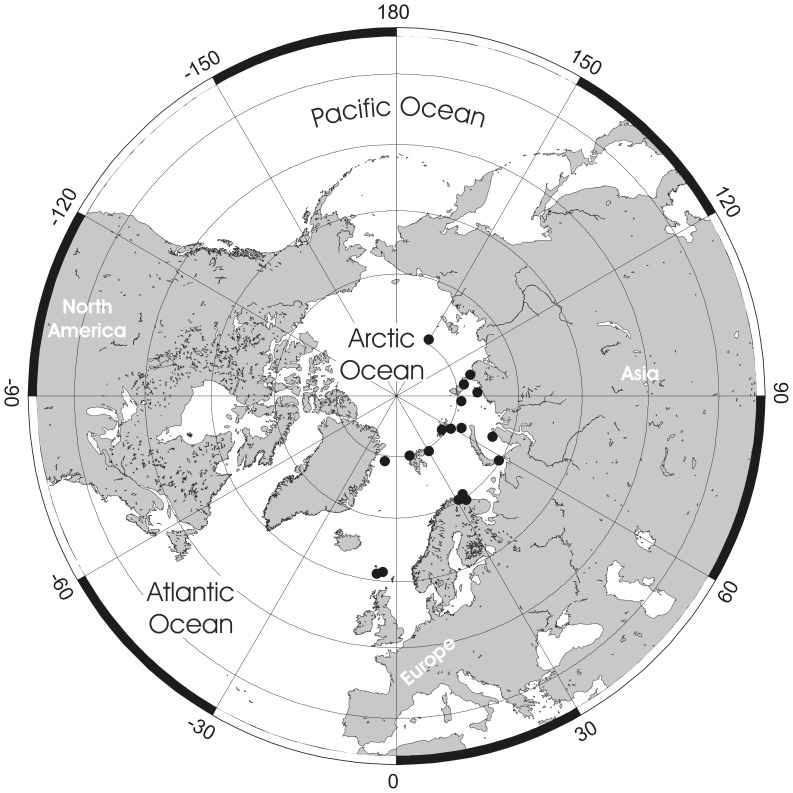
Distributional records of *Pseudoflustra anderssoni* gathered from the literature and museum collections. (circles – record confirmed either by scanning electron or light microscopy, squares – unconfirmed record taken from literature). Note that some symbols represent more than one record as the resolution of the map is insufficient to depict all records.

This is a North Atlantic–Arctic species occurring between 100 and 1000 m depth, and 11°W and 150°E, including Greenland, the Barents, Kara, Laptev and East Siberian seas, and the Gulf of St. Lawrence. The lowest recorded latitude is 61°N (Figure 7).

**Figure 8:**
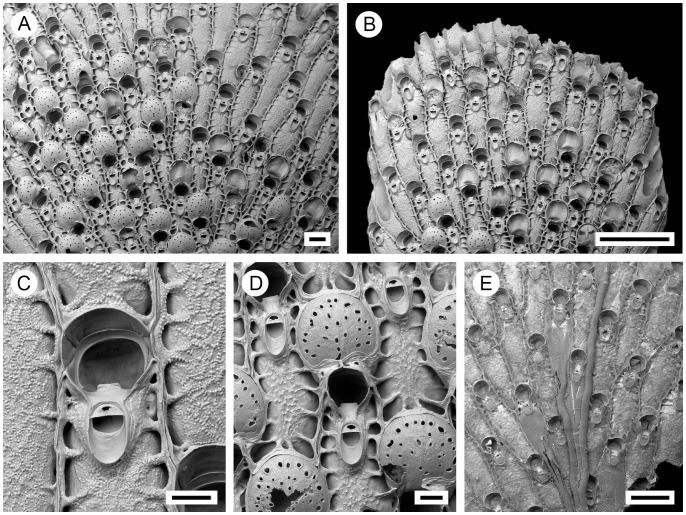
*Pseudoflustra sinuosa* (Andersson, 1902). A. colony showing autozooids and ovicellate zooids, bleached; B. distal colony edge with autozooids and ovicellate zooids, bleached; C. orifice of autozooid with avicularium, bleached; D. ovicellate zooid with suboral avicularium, bleached; E. characteristic chitinized cuticular rhizoids growing proximally as inflations of frontal cuticles of autozooids, unbleached. A, B, C, E, NHM 11.10.1. 1498A; D, NHM 2012.3.7.5. Scale bars: 1 mm (B), 500 µm (E), 200 µm (A) 200 µm (C, D).

***Pseudoflustra sinuosa*** (Andersson, 1902)

(Figures 8A–E, Table 1)

*Smittina palmata* var. *sinuosa* Andersson, 1902: 546, pl. 30, fig. 5 [38].

*Pseudoflustra sinuosa* (Andersson) – Kluge, 1962: 445, fig. 295 [30]; Kluge, 1975: 539, fig. 295 [25].

### Type Material

Holotype: Uppsala Museum no 121.

### Other Material Examined

See Appendix S1.

### Description

Colony erect, flat, bifoliate, branches widening distally, sometimes bifurcating; basally rooted, the cuticular rootlets originating through expansion of autozooidal frontal cuticles, initially adherent to branch surface then clustering to form thick bundles descending into soft substrate (Figure 1, 8E). Ancestrula unknown.

Autozooids arranged in straight longitudinal rows, with up to 21 rows on each branch side, slender, oblong-rectangular, often narrowing proximally, separated by shallow grooves, large. Frontal shield granular, granules sometimes arranged in a polygonal network; areolar pores conspicuous, oval, elongated-oval or irregular, often with thickened ridges, opening towards centre of shield, sometimes occupying large part of frontal shield (Figures 8A, B). Primary orifice round, slightly longer than wide, condyles at proximolateral corners, denticle located medially on proximal margin sometimes expanding towards end; secondary orifice oval, cormidial, peristome pseudosinuate, distally and sometimes partly laterally constituted by calcification of neighbouring zooids, lateral lobes of peristome enclosing suboral avicularium (Figures 8C–D). Ovicell hyperstomial, round, globular, slightly broader than long, large, lateral and distal borders overgrown by calcification of neighbouring zooids, overgrowths from left and right sides sometimes fusing to form a bridge across proximal part of ovicell; ectooecium pseudoporous, pores numbering 17 to 24 (mean 20, n = 20), variously shaped, scattered over entire surface, outer row of pores rather regular and radially elongated, inner pores irregular (Figure 8D).

Avicularia adventitious, suboral, oval, large, longer than wide, directed proximally; opesia small, semielliptical; rostrum elongated elliptical with parallel lateral margins, distal calcified shelf extensive; mandible elongated, ligulate; crossbar calcified, broad; space between orifice and avicularium occupied by avicularian chamber which spreads to proximal corners of orifice (Figures 8C–D).

### Remarks

Although we have not seen the type specimen of *Pseudoflustra sinuosa*, the detailed original description and drawing given by Andersson [38] permits a confident determination. Both the autozooidal and orificial dimensions of *P. sinuosa* are the largest among the known species of *Pseudoflustra*. Furthermore, this is the only species of *Pseudoflustra* in which the orifice has a denticle that is very similar to a lyrula in morphology. Of the three other species having a denticle, this structure is most pronounced in *P. perrieri* but rather inconspicuous in *P. minima* and *P. birulai*.

### Distribution

**Figure 9:**
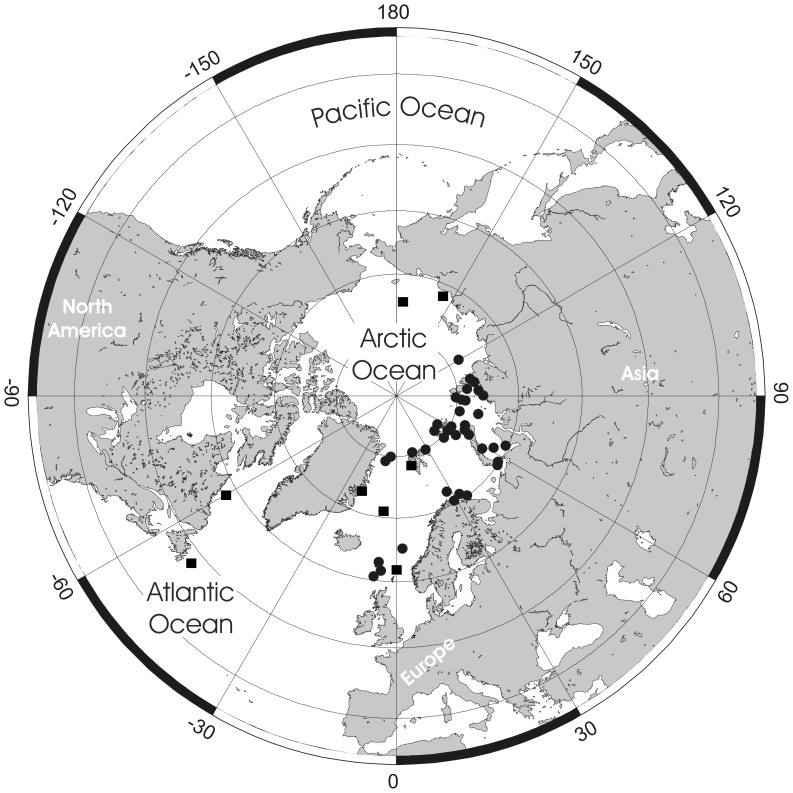
Distributional records of *Pseudoflustra sinuosa* gathered from the literature and museum collections. (circles – record confirmed either by scanning electron or light microscopy, squares – unconfirmed record taken from literature). Note that some symbols represent more than one record as the resolution of the map is insufficient to depict all records.

This is an Arctic species occurring at depth of 100–1000 m, and with confirmed records between 7°W and 172°W, including Greenland and the Barents, Kara, Laptev East Siberian and Chukchi seas [25], [39]. The lowest confirmed latitude is 60°N (Figure 9). Powell’s [13] material from 50°N, 48°W requires confirmation; Kluge’s specimen from the East Siberian Sea could not be located in his collection.

**Figure 10:**
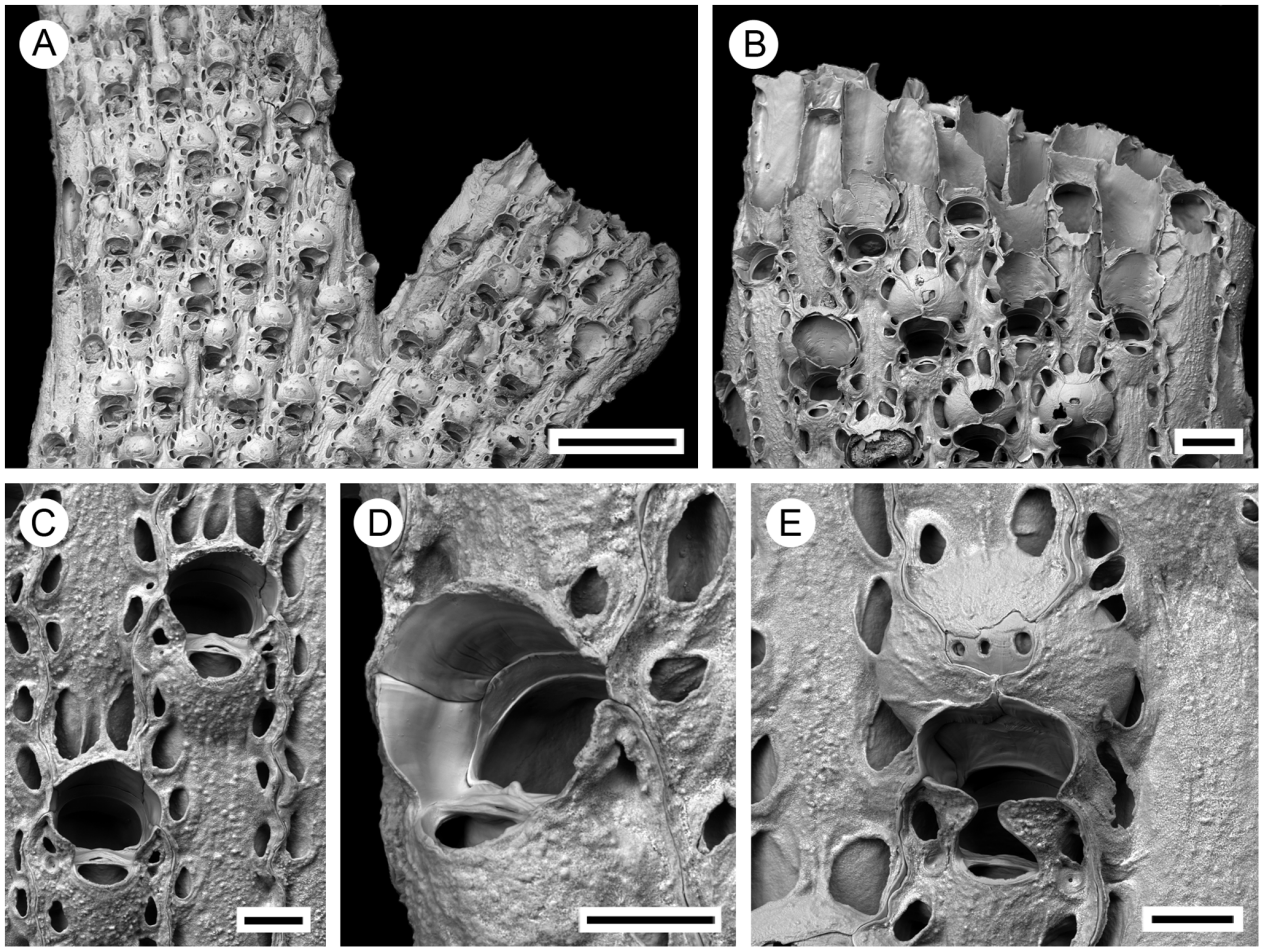
*Pseudoflustra birulai* Kluge, 1929. A. colony showing autozooids and ovicellate zooids, bleached; B. distal colony edge with autozooids and ovicellate zooids, bleached; C. orifice of autozooids with avicularia, bleached; D. side view of orifice of autozooid with avicularium, bleached; E. ovicellate zooid with suboral avicularium, bleached. A, ZI RAS 9; B, C, D, E, ZI RAS 12; Scale bars: 1 mm (A), 200 µm (B), 100 µm (C, D, E).

***Pseudoflustra birulai*** Kluge, 1929

(Figures 10A–E, Table 1)

*Pseudoflustra birulai* Kluge, 1929: 16–17 [40]; Kluge, 1946: 199, pl. 2, fig. 3 [36]; Kluge, 1962: 446, fig. 296 [30]; Kluge, 1975: 540, fig. 296 [25]; Gontar & Denisenko, 1989, pl. 14-IV, fig. 5 [31].

### Type Material

Lectotype (here designated): ZI 2, Tajmyr Bay, Stn 44, 76°59.5′N, 100°19.5′E, *Zaria*, 1901, collected by Russian Polar Expedition under conduction of baron Toll. This specimen is here designated as the lectotype based on the match between the locality information and that given in the original description [40].

### Other Material Examined

See Appendix S1.

### Description

Colony erect, flat, bifoliate, wider towards the distal end, sometimes bifurcating; basally rooted, each cuticular rhizoid growing proximally as an inflation of the frontal cuticle of an autozooid, initially adhering to colony surface before clustering into thick bundles that enter the substrate (Figure 1). Ancestrula unknown.

Autozooids arranged in straight rows, up to 17 on each side of branch, slender, oblong-rectangular, separated by shallow grooves, often narrowing proximally, large. Frontal shield granular; areolar pores conspicuous, large, oval, elongated-oval or irregular, often with thickened ridges facing inward, alveoli sometimes minute (in older zooids) but may appear to occupy all frontal shield (Figures 10A, B). Primary orifice round, slightly wider than long, with smooth suboral shelf ending in condyles at proximolateral corners, proximal margin straight with a small sharp denticle in the middle; secondary orifice oval, cormidial, in ovicellate zooids personate, lappets forming bridge over secondary orifice with proximal pseudosinus (Figures 10C, E). Ovicell hyperstomial, round, globular, slightly broader than long, large, largely overgrown at the edges by calcification of surrounding zooids; pores numbering 1 to 4 (mean 3, n = 20), varying in shape and scattered irregularly over whole surface (Figures 10B, E).

Avicularia adventitious, oval, deeply immersed in secondary orifice, steeply inclined and not always visible, wider than long; rostrum semielliptical, distal calcified shelf lacking; crossbar calcified; avicularian chamber raised above frontal shield of host autozooid, becoming lower towards proximal end (Figures 10C, D).

### Remarks

*Pseudoflustra birulai* differs from other species of the genus in having a very distinctive avicularium inclined at a high angle to the frontal shield and therefore not fully visible in frontal view. In addition, the avicularium has no distal calcified shelf, a feature shared only with *P. solida*, and the peristomial lappets overarching the orifice are very pronounced.

### Distribution

**Figure 11:**
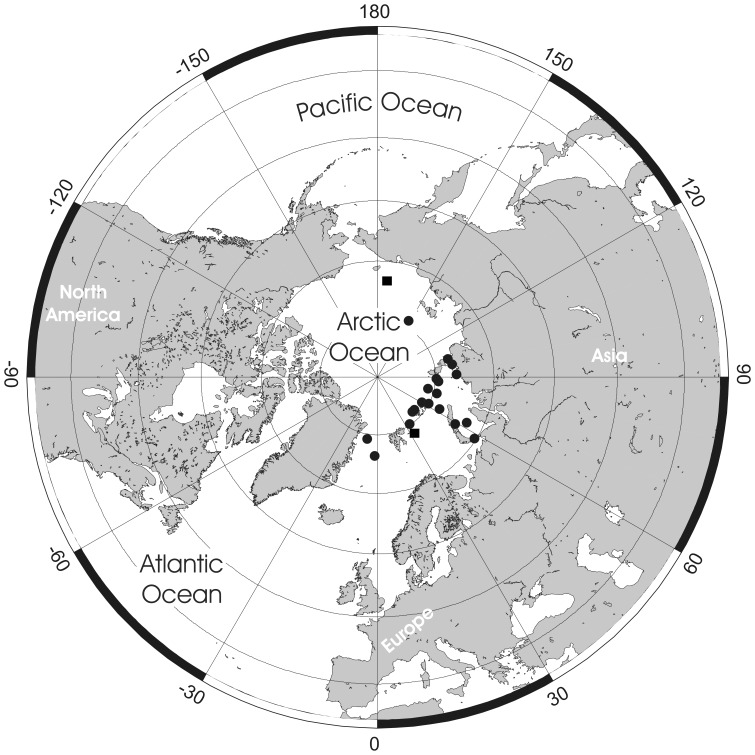
Distributional records of *Pseudoflustra birulai* gathered from the literature and museum collections. (circles – record confirmed either by scanning electron or light microscopy, squares – unconfirmed record taken from literature). Note that some symbols represent more than one record as the resolution of the map is insufficient to depict all records.

This is an Arctic species occurring between 90 and 700 m depth, and 12°W and 172°W, including Greenland and the Barents, Kara, Laptev, East Siberian and Chukchi seas [24], [39], [41]. The lowest recorded latitude is 70°N (Figure 11).

**Figure 12:**
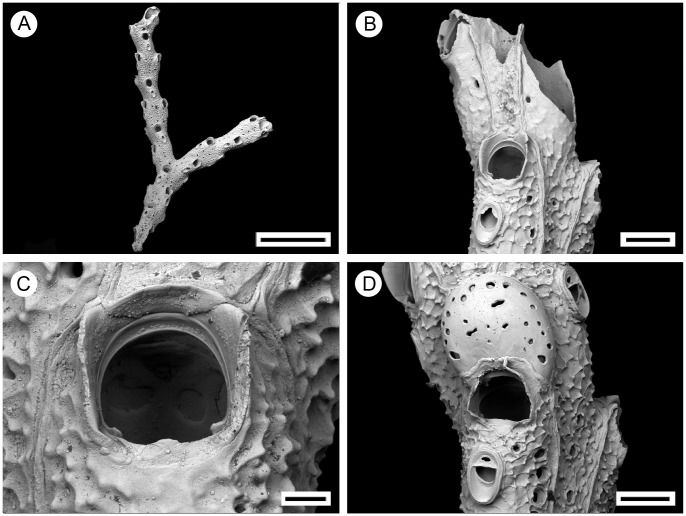
*Pseudoflustra minima* Hayward, 1994, Holotype: ZM 17, bleached. A. colony showing autozooids and ovicellate zooids; B. distal colony edge with autozooids; C. orifice of autozooids; D. ovicellate zooid with suboral avicularium. Scale bars: 1 mm (A), 100 µm (B, D), 30 µm (C).

***Pseudoflustra minima*** Hayward, 1994

(Figures 12A–D, Table 1)

*Pseudoflustra minima* Hayward, 1994: 202, fig. 11D, E [15].

### Type Material

Holotype: ZM 17, BIOFAR stn 489, 23.07.1989, 61.3367°N, 10.1983°W; 1200 m depth, detritus sledge, temp. 4°C, salinity 35.2 psu. Paratype: ZM 18, details as for holotype.

### Other Material Examined

See Appendix S1.

### Description

Colony erect, vinculariiform, bifurcating. Some basal zooids have inflated cuticles forming tubes adhering to colony surface and united as rhizoidal bundles entering the substrate (Figures 1, 12A). Ancestrula unknown.

Autozooids arranged circumferential in three or four rows, slender, oblong-rectangular, separated by shallow grooves, often narrowing towards proximal end. Frontal shield covered by polygonal ridges, triple junctions between the confluent ridges raised; areolar pores variable in size (Figures 12A, B). Primary orifice round, with smooth suboral shelf ending in condyles at proximolateral corners, proximal margin straight with small, sharp median denticle; secondary orifice oval, cormidial, with peristome developed laterally; in ovicellate zooids peristome developed around entire orifice except for an opening in middle of proximal side, lateral lobes of peristome stretching above both sides of distal part of avicularium and fusing in the middle (Figures 12B, D). Ovicell hyperstomial, globular, slightly broader than long, encapsulated by surrounding zooids, pseudopores numbering 24 (n = 1), varying in shape, scattered irregularly over entire surface (Figure 12D).

Avicularia adventitious, oval, suboral, longer than wide, directed proximally, distal calcified shelf extensive, rostrum triangular with round tip, mandible elongated, crossbar calcified, ligulate, broad (Figure 12D).

### Remarks

This species differs from all other species of *Pseudoflustra* by a combination of several characters. Firstly, *P. minima* has a delicate vinculariiform colony, a character shared with other temperate species of *Pseudoflustra* (*P. virgula*, *P. radeki* sp. nov., *P. perrieri*). However, autozooids in *P. radeki* sp. nov. are distributed only frontally, whereas in *P. minima* they occur circumferentially. Additionally, *P. minima* has a frontal shield with a very pronounced polygonal patterning. This is absent in all other species of *Pseudoflustra* apart from *P. sinuosa*, in which the polygons formed by granules are much less conspicuous than those of *P. minima*. Finally, the orifice in *P. minima* possesses a minute denticle. This character is shared only with two other species, *P. birulai* and *P. perrieri*, the former having bifoliate colonies much larger than those of *P. minima* and a strikingly different avicularium that is hidden within the secondary orifice, and the latter possessing triangular avicularia contrasting with the oval avicularia of *P. minima*.

### Distribution

**Figure 13:**
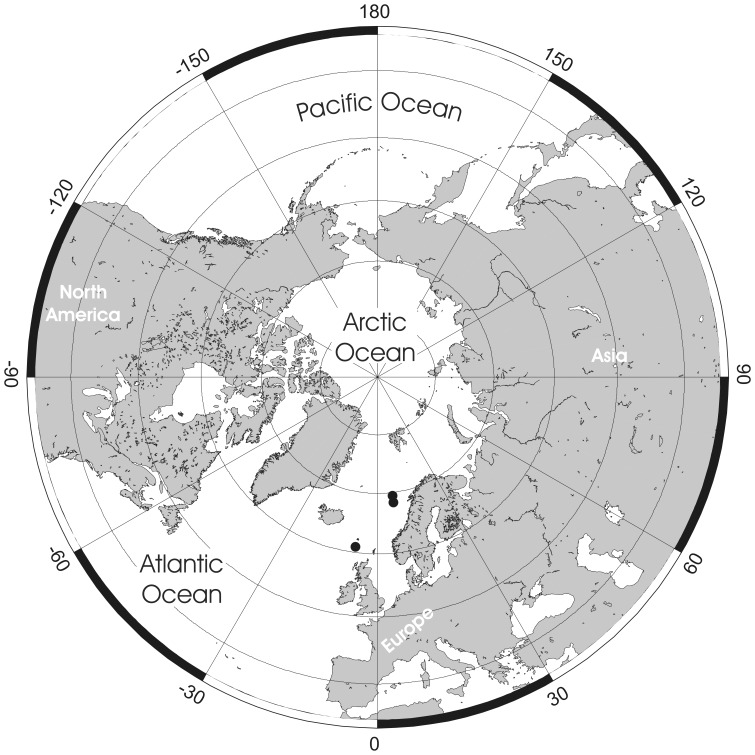
Distributional records of *Pseudoflustra minima* gathered from the literature and museum collections. (circles – record confirmed either by scanning electron or light microscopy, squares – unconfirmed record taken from literature). Note that some symbols represent more than one record as the resolution of the map is insufficient to depict all records.

Colonies of *Pseudoflustra minima* have been recorded at the Faroes [15] and in the Norwegian Sea (present study) (Figure 13), ranging from 200 to 1200 m water depth. Therefore, the known distribution of the species is temperate northeastern Atlantic.

**Figure 14:**
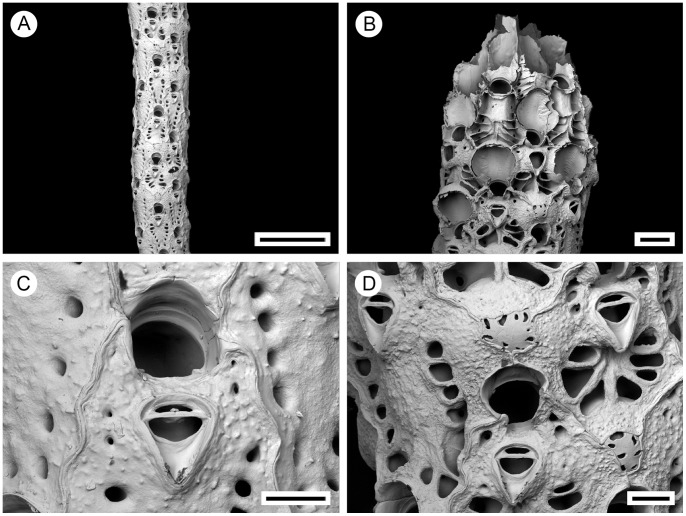
*Pseudoflustra virgula* Hayward, 1994, Holotype: ZM 19, bleached. A. colony showing autozooids and ovicellate zooids; B. distal colony edge with autozooids and ovicellate zooids; C. orifice of autozooid with avicularium; D. ovicellate zooid with suboral avicularium. Scale bars: 1 mm (A), 200 µm (B), 100 µm (C, D).

***Pseudoflustra virgula*** Hayward, 1994

(Figures 14A–D, Table 1)

*Pseudoflustra virgula* Hayward, 1994: 202, fig. 12A–D [15].

### Type Material

Holotype: ZM 19, BIOFAR, stn 736, 30.09.1990, 61.2843°N, 10.5345°W, 1157 m depth, detritus sledge, temp. 6.5°C, salinity 35.2 psu. Paratype: ZM 118, BIOFAR stn 489, 61.3367°N, 10.1983°W, 1200 m depth, 23.07.1989.

### Other Material Examined

See Appendix S1.

### Description

Colony erect, vinculariiform, slightly wider towards the distal end, all studied material unbranched. Basal zooids forming cuticular rhizoids growing proximally along colony surface before clustering into bundles descending into the substrate (Figures 1, 14A). Ancestrula unknown.

Autozooids arranged circumferentially in 7–8 alternating, straight longitudinal rows, oblong-hexagonal to oblong-irregular, often narrowing proximally, separated by shallow grooves. Frontal shield granular, areolae conspicuous, large, oval, pores elongate oval or irregular, seeming to occupy all frontal shield in young zooids, minute in old zooids (Figures 14A, B). Primary orifice round, with suboral smooth shelf ending in condyles at the proximolateral corners, proximal margin straight; secondary orifice oval, cormidial, especially in ovicellate zooids, low peristome developed around proximolateral parts of orifice with opening in the middle of proximal side, lateral lobes of peristome stretching towards avicularium (Figures 14C, D). Ovicell hyperstomial, globular, slightly broader than long, encapsulated and largely overgrown at edges by calcification of surrounding zooids, opposite lobes of calcification at proximal end often fused, pseudopores numbering 7 to 10 (mean 8, n = 6), variously shaped, scattered irregularly over whole surface (Figure 14D).

Avicularia adventitious, suboral, directed proximally, triangular, longer than wide, rostrum triangular with pointed tip, mandible elongated, crossbar calcified, ligulate, distal calcified shelf extensive (Figures 14C, D).

### Remarks

As Hayward [15] stated in the original description, *Pseudoflustra virgula* differs strikingly from other species of *Pseudoflustra* in its suboral avicularium which is triangular and pointed distally. All other species, except for *P. perrieri*, have oval avicularia. Although *P. perrieri* has avicularia similar in shape to *P. virgula*, *P. perrieri* has a very distinct denticle on the proximal margin of the primary orifice absent in *P. virgula.* Concerning zooid morphology, *P. virgula* is also closely related to *Smittia azorensis* Jullien in Jullien & Calvet, 1903 (p. 99, pl. 11, figure 5) [42]. However the latter species, which certainly belongs to the genus *Pseudoflustra*, forms broadly bilaminar colonies, and is therefore considered as a distinct species. Material of *P. azorensis* could not be located at the MNHN Paris (it is presumably at the Oceanographic Museum Monaco), and was not included in the present study.

### Distribution

**Figure 15:**
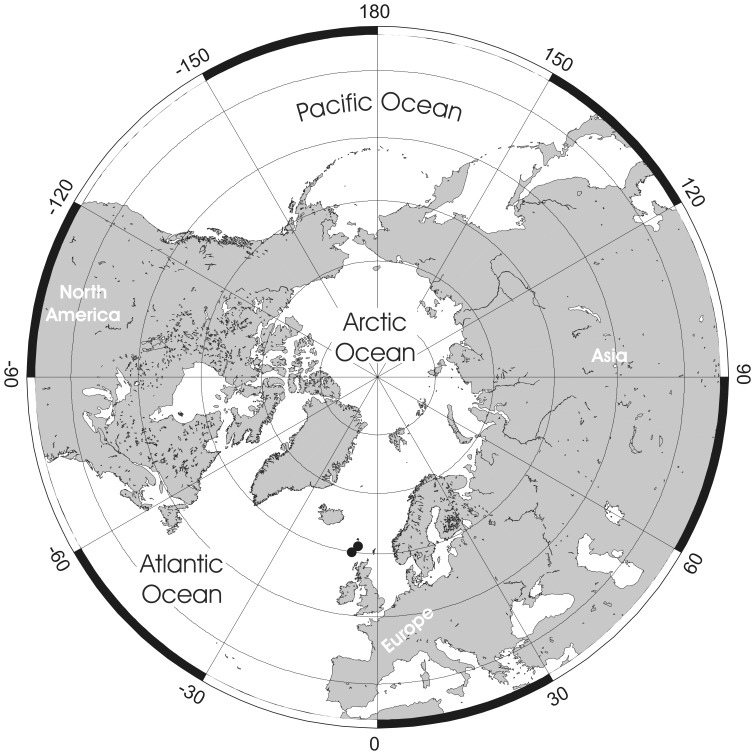
Distributional records of *Pseudoflustra virgula* gathered from the literature and museum collections. (circles – record confirmed either by scanning electron or light microscopy, squares – unconfirmed record taken from literature).

*Pseudoflustra virgula* has been recorded from four locations, all off the Faroe Islands (Figure 15), ranging from 914 to 1200 m in depth. It is a temperate northeastern Atlantic species.

**Figure 16:**
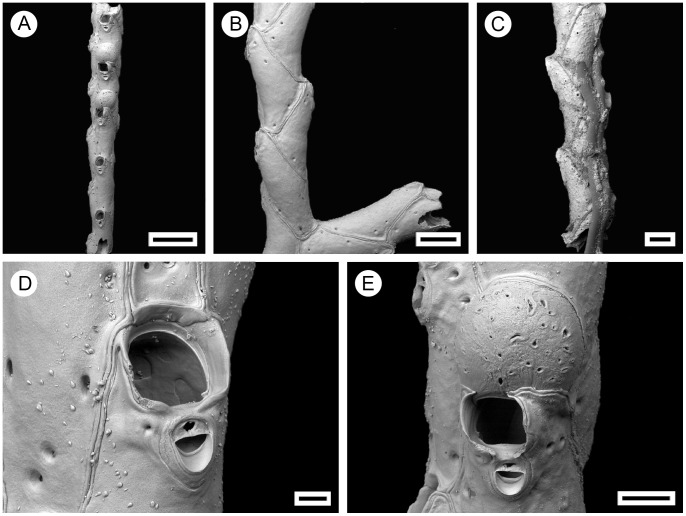
*Pseudoflustra radeki* sp.nov. A. colony showing autozooids and ovicellate zooids, bleached; B. basal view of the colony, bleached; C. basal view of the colony with characteristic chitinized cuticular rhizoids growing from basal side of autozooids, unbleached; D. orifice of autozooid with avicularium, bleached; E. ovicellate zooid with suboral avicularium, bleached. A, B, D, E, MNHN 7206; C, MNHN 7209. Scale bars: 500 µm (A), 200 µm (B, C), 100 µm (E), 30 µm (D).

***Pseudoflustra radeki*** sp. nov.

urn:lsid:zoobank.org:act:26EE98CA-CDA4-43C1-80DE-08E0E65C77C0.

(Figures 16A–E, Table 1)

Not *Ichthyaria aviculata* Calvet, 1906: 215 [43]; 1907: 414, pl. 27, figs 1–2 [44].

*Pseudoflustra aviculata* (Calvet) – d’Hondt, 1973: 380 [14].

### Etymology

Named after Radek Szczęch, who died suddenly in 2010, for his friendship.

### Type Material

Holotype: MNHN 7206, *Thalassa*, stn 415, 40°34.3′N, 08°41.7′W, 450 m depth, det. J.-L. d’Hondt. Paratype: MNHN 7209, details as for holotype.

### Other Material Examined

*Ichthyaria aviculata* Calvet, 1906, MNHN 3765, holotype by monotypy, *Talisman*, stn 96, 15.7.1883, 19°19′N, 18°02′W, 2330 m depth, off Mauritania.

### Description

Colony erect, vinculariiform, bifurcating, formed of separated basal parts connected by cuticular tubes, zooids opening only on frontal surface. Basal zooids forming cuticular rhizoids growing proximally along colony surface before clustering into bundles descending into substrate (Figures 1, 16A–C). Ancestrula unknown.

Autozooids arranged frontally in two straight, alternating, longitudinal rows, oblong-hexagonal, separated by shallow grooves. Frontal shield and abfrontal surface smooth, with few, small, irregularly scattered areolar pores along lateral edges (Figures 16A, D). Primary orifice round, slightly wider than high, with smooth suboral shelf ending in short triangular condyles at the proximolateral corners, proximal margin straight; secondary orifice oval, cormidial; peristome especially well developed in ovicellate zooids around orifice, with opening in the middle proximal side of orifice, lateral lobes of peristome partly enclosing the proximal edge of ovicell and stretching towards the avicularium (Figures 16D, E). Ovicell hyperstomial, round, globular, slightly broader than long, edges encapsulated by surrounding zooids, pseudopores numbering 26 to 28 (n = 2), varying in shape, scattered irregularly over entire surface (Figures 16E).

Avicularia adventitious, suboral, oval, longer than wide, directed proximally, palate semielliptical, mandible elongated, crossbar calcified, ligulate, distal calcified shelf extensive; avicularian chamber raised above the frontal shield (Figures 16D, E).

### Remarks

This species was synonymised with *Ichthyaria aviculata* Calvet, 1906 and assigned to *Pseudoflustra* by d’Hondt [14]. The specimens used for our description do not match the locations of specimens analysed by d’Hondt [14], which could not be found in the MNHN collection. However, as they are from the same area, similar depths, and were determined by the same taxonomist (J.-L. d’Hondt), we presume that they are conspecific.

The holotype of *I. aviculata* (MNHN 3765) lacks ovicells and is too poorly preserved for a thorough redescription. However, it is apparently not the same species as that described under this name by d’Hondt [14]. A new species name is therefore required. The type specimen, as well as the original figure, of *I. aviculata* shows a frontal shield with a very conspicuous polygonal ornamentation resembling that of *P. minima* (Figures 12B–D). In contrast *P. radeki* sp. nov. has a smooth frontal shield. In addition, *I. aviculata* lacks the areolar pores along zooidal margin which are present in *P. radeki* sp. nov.

The new species differs from all other species of *Pseudoflustra* in having vinculariiform colonies with autozooidal orifices opening only on the frontal surface. In other species with vinculariiform colonies (*P. minima*, *P. virgula*, *P. perrieri*) autozooidal orifices are distributed circumferentially.

### Distribution

**Figure 17:**
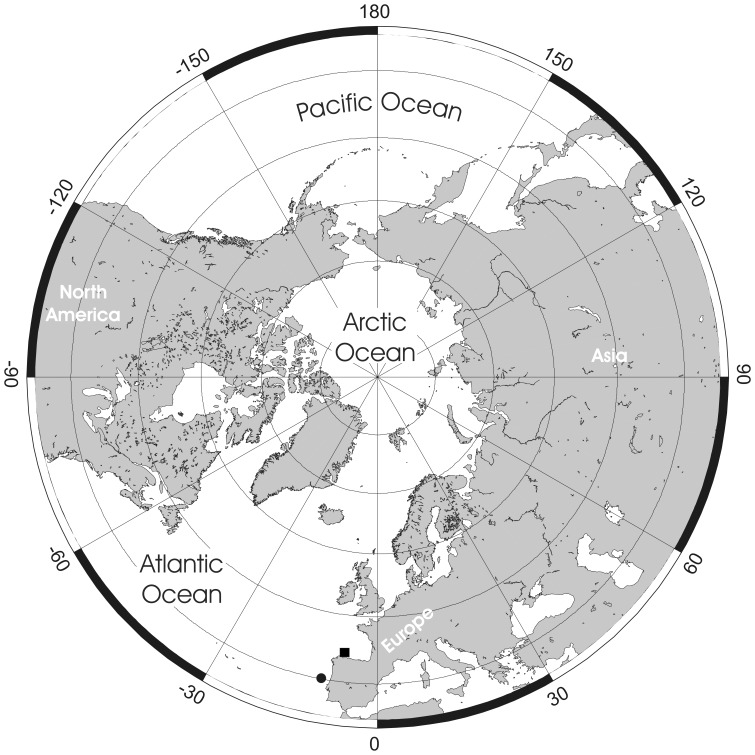
Distributional records of *Pseudoflustra radeki* sp.nov. gathered from the literature and museum collections. (circles – record confirmed either by scanning electron or light microscopy, squares – unconfirmed record taken from literature). Note that some symbols represent more than one record as the resolution of the map is insufficient to depict all records.

*Pseudoflustra radeki* sp. nov. has been recorded from three locations, ranging in depth from 450 to 630 m, all along the Iberian Peninsula (Figure 17). Therefore, its distribution can be characterized as temperate northeastern Atlantic.

**Figure 18:**
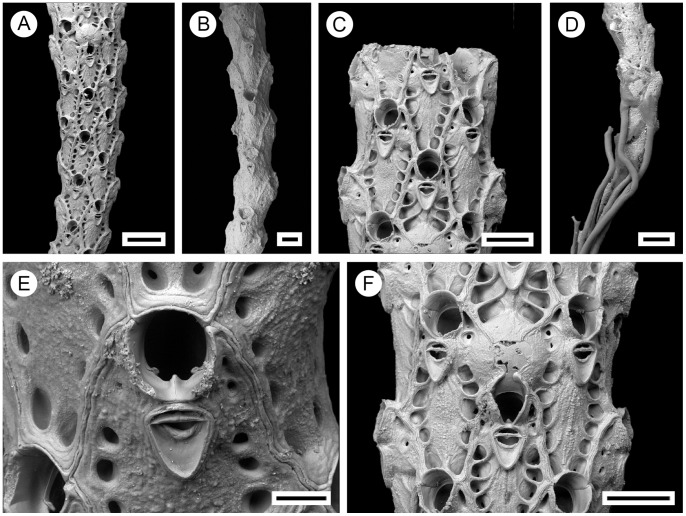
*Pseudoflustra perrieri* (Jullien, 1883). A. colony showing autozooids and ovicellate zooids, bleached; B. overview of lectotype colony showing autozooids, unbleached; C. distal colony edge with autozooids and ovicellate zooids, bleached; D. basal view of the colony with characteristic chitinized cuticular rhizoids growing from basal side of autozooids, unbleached; E. orifice of autozooid with avicularium, bleached; F. autozooids and ovicellate zooid with suboral avicularia, bleached. A, C, E, F, NHM 2002.9.10.16; B MNHN 1693; D, NHM 1999.6.23.4. Scale bars: 500 µm (A), 300 µm (C, D, F), 200 µm (B) 100 µm (E).

***Pseudoflustra perrieri*** (Jullien, 1883)

(Figures 18A–F, Table 1)

*Smittia perrieri* Jullien, 1883: 515, pl. 16, fig. 45 [45]; d'Hondt, 1975: 578 [46].

*Smittoidea perrieri* (Jullien) – Hayward, 1978: 217, fig. 4a, b [25].

### Material

Lectotype (here designated): MNHN 1693, 43°0.7′N, 11°57.7′W, 2018 m depth, 13.6.1881, *Travailleur*, stn 1; this specimen is the only one from the sampling station originally recorded by Jullien [45] that is present in the Paris collection. It was suggested by Tricart & d’Hondt [47] to serve as type, and we here formally designate this specimen as lectotype.

### Other Material Examined

MNHN 3789, Bay of Biscay (exact location unknown), 1353 m, *Travailleur* (date unknown), stn 6; NHM 1999.6.23.4, 47°33.8′N, 07°12.6′W, 511 m depth, 22.10.1973, *Thalassa*, stn Z 397, det. P.J. Hayward; NHM 2002.9.10.16, 47°36′N, 07°16.8′W, 330 m depth, 22.10.1973, *Thalassa*, Z 398, det. P.J. Hayward.

### Description

Colony erect, vinculariiform, examined material unbranched. Basal zooids forming cuticular rhizoids growing proximally along colony surface before descending into substrate (Figures 1, 18A, B, D). Ancestrula unknown. Autozooids arranged circumferentially in 4–8 straight, alternating, longitudinal rows, zooids oblong hexagonal, separated by distinct sutures.Frontal shield granular; areolae arranged irregularly along edges, areolar pores large, becoming deeply inset and separated by ridges in older zooids (Figures 18A, C). Primary orifice round, slightly longer than wide, with a smooth suboral shelf; conspicuous condyles at the proximolateral corners; sinus shallow with sharp denticle in the middle; secondary orifice oval, cormidial, peristome developed around orifice especially proximolaterally (Figure 18E). Ovicell hyperstomial, globular, slightly broader than long, large, edges encapsulated by secondary calcification of surrounding zooids, pseudopores numbering 4 to 5 (n = 2), varying in shape, scattered irregularly over entire surface (Figure 18F).

Avicularia adventitious, suboral, longer than wide, directed proximally, rostrum triangular, tip acute, distal calcified sheld extensive, crossbar calcified, curved; avicularian chamber slightly raised above the frontal shield (Figures 18E–F).

### Remarks

*Pseudoflustra perrieri* was first assigned to the genus *Smittia* by Jullien (1883) and later moved into *Smittoidea* by Hayward [26]. However, Hayward [26] noted uncertainty over the generic attribution of the species. Several characters support its reassignment to *Pseudoflustra*. Firstly, *P. perrieri* has an erect growth form like other species of *Pseudoflustra* but unlike *Smittoidea*, which is generally encrusting. Colonies of *P. perrieri* are attached to the substrate by means of cuticular rootlets, as is typical for *Pseudoflustra*. The cormidial secondary orifice is also typical of *Pseudoflustra*. Finally, there are no oral spines present in any ontogenetic or astogenetic stage of *P. perrieri*, whereas oral spines are a rather common feature in *Smittoidea*.

Only two species of *Pseudoflustra* have suboral avicularia with triangular rostra, *P. perrieri* and *P. virgula*. They can be distinguished by the presence of a denticle on the proximal margin of the primary orifice in *P. perrieri*, which contrasts with the straight proximal margin in *P. virgula*.

The number of longitudinal autozooidal rows differs within and between colonies. Proximal parts are usually composed of only four rows whereas there may be eight rows in distal parts. The small lectotype entirely consists of four series (Figure 18B), which may therefore represent a proximal colony part and/or an ecophenotype as the lectotype was found at considerably greater depths than the remaining specimens.

### Distribution

**Figure 19:**
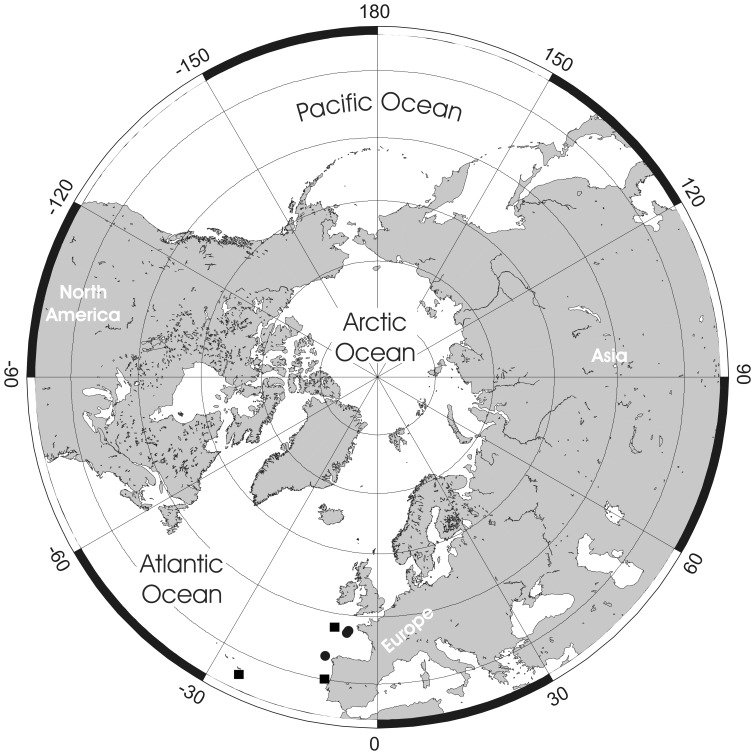
Distributional records of *Pseudoflustra perrieri* gathered from the literature and museum collections. (circles – record confirmed either by scanning electron or light microscopy, squares – unconfirmed record taken from literature).

This species has been recorded from six localities (plus one of unknown exact location) in the Bay of Biscay, northwestern Iberia, and the Azores (Figure 19), ranging in depth from 330 to 2018 m. Therefore it can be characterized as a temperate northeastern Atlantic species.

### Phylogenetic Analysis

A cladistic analysis was undertaken to infer phylogenetic relationships between the nine species of *Pseudoflustra* particularly in the context of phylogeography. A matrix of 29 morphological characters was assembled (Table 2). These could be fully coded for all nine species (i.e. without questionable character states), plus an outgroup species.

**Table 2:** Morphological characters and character states used in the phylogenetic analysis.

1. colony-form: (0) bifoliate; (1) vincularian; (2) encrusting						
2. autozooid orifice distribution: (0) circumferential; (1) frontal only						
3. denticle/lyrula: (0) absent; (1) denticle; (2) lyrula						
4. orifice length: (0) <0.16 mm; (1) >0.16 mm						
5. orifice width: (0) <0.16 mm; (1) >0.16, <0.19 mm; (2) >0.19 mm						
6. orifice length/width: (0) <0.80; (1) >0.80, <0.98; (2) >0.98						
7. lappets: (0) absent; (1) present						
8. cryptocyst between orifice and avicularium: (0) absent; (1) present						
9. cormidial secondary orifice: (0) absent; (1) present						
10. polygons on frontal shield: (0) absent; (1) present						
11. autozooid length: (0) <0.89 mm; (1) >0.89, <1.10 mm; (2) >1.10 mm						
12. autozooid width: (0) <0.32 mm; (1) >0.32, <0.39 mm; (2) >0.39 mm						
13. autozooid length/width: (0) <3; (1) >3, <4; (2) >4						
14. autozooid length/orifice length: (0) <7; (1) >7, <9; (2) >9						
15. ovicell pore number: (0) <10; (1) >10, <20; (2) >20						
16. ovicell length: (0) <0.24 mm; (1) >0.24, <0.36 mm; (2) >0.36 mm						
17. ovicell width: (0) <0.30 mm; (1) >0.30, <0.39 mm; (2) >0.39 mm						
18. ovicell length/width: (0) <0.80; (1) >0.80, <0.92; (2) >0.92						
19. overgrowth of ovicell: (0) absent; (1) partial; (2) complete						
20. avicularium shape: (0) oval; (1) triangular						
21. avicularium inclination (0) acute to frontal; (1) high angle to frontal						
22. avicularium length: (0) <0.13 mm; (1) >0.13, <0.23 mm; (2) >0.24 mm						
23. avicularium width: (0) <0.10 mm; (1) >0.10, <0.15 mm; (2) >0.15 mm						
24. avicularium length/width: (0) <0.95; (1) >0.95						
25. palate: (0) absent; (1) present						
26. distance between orifice and avicularium: (0) = 0; (1) >0 mm						
27. autozooid length/avicularium length: (0) <8.20; (1) >8.20						
28. proximo-lateral suboral avicularium: (0) absent; (1) present						
29. oral spines: (0) absent; (1) present						

Fifteen of the 29 characters are multistate, the remainder binary. Of the 16 quantitative characters, 6 are proportions, previously found to be useful in improving resolution during cladistic analyses of cheilostome bryozoans [48], [49]. Gap analysis was used to determine character states for quantitative characters.

**Figure 20:**
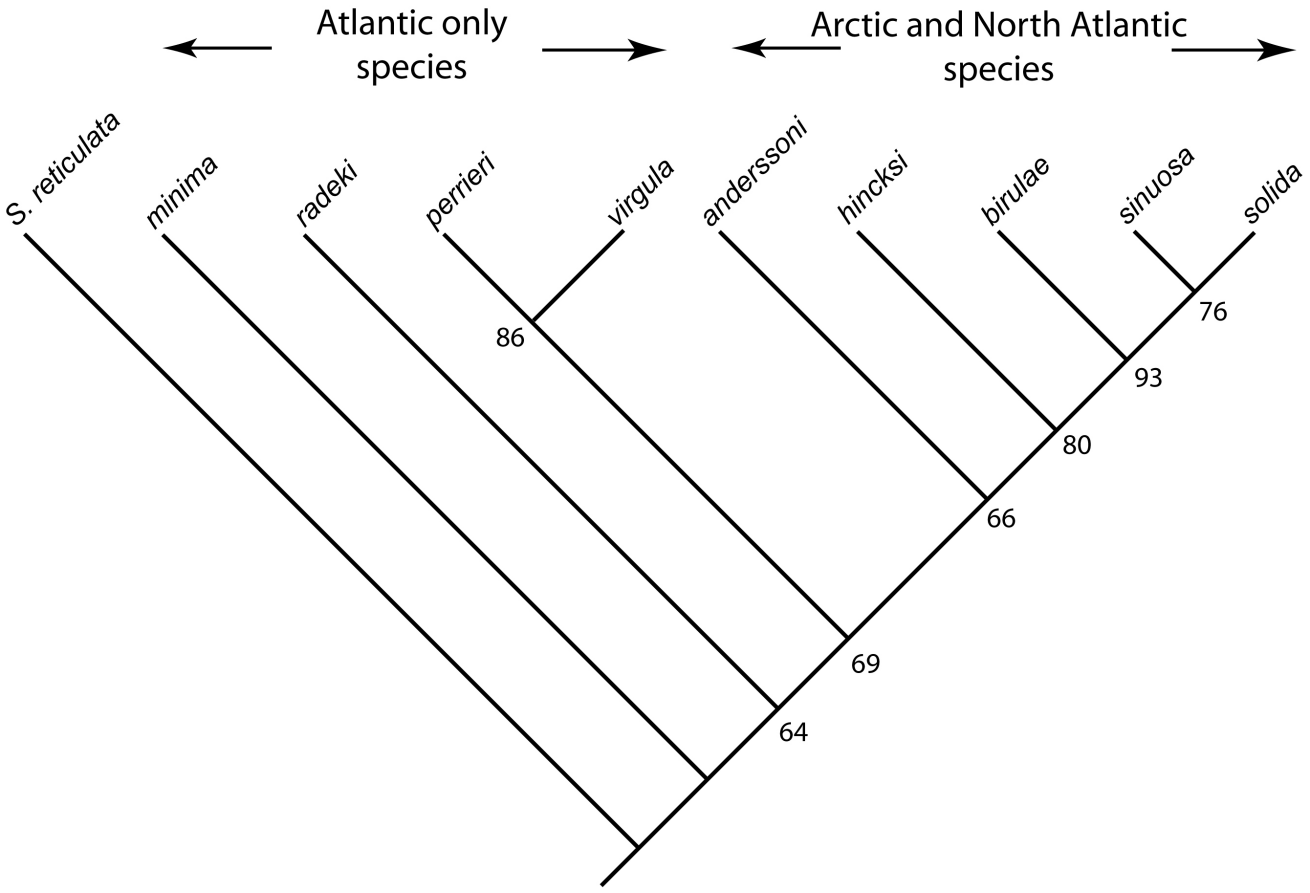
Phylogenetic relationships between the nine species of *Pseudoflustra* based on a cladistic analysis. Tree shown is the single, most parsimonius tree after reweighting of characters based on their rescaled consistency indices, and is rooted on *Smittoidea reticulata*. Numbers at nodes are bootstrap support values.

The analysis was run using PAUP 4.0. Three equally parsimonius trees, length 77 steps, were found after an exhaustive search. Characters were next reweighted according to the maximum value of the rescaled consistency index and the analysis was re-run. The single resulting tree (Figure 20) has a consistency index (*CI*) of 0.748, retention index (*RI*) of 0.712 and rescaled consistency index (*RC*) of 0.533.

Outgroup selection is problematical, as is so often the case for bryozoans due to the lack of an existing phylogenetic framework. We chose *Smittoidea reticulata* (MacGillivray, 1842) as the outgroup because of its close resemblance to *Pseudoflustra*, a similarity that has been recognised for more than a century [21] (Table 3). This species has been recorded from the Barents Sea and the eastern Atlantic as far south as Morocco and the western Mediterranean [50].

**Table 3:** Character matrix for the 9 species of *Pseudoflustra* plus the outgroup species, *Smittoidea reticulata*.

		10	20
*P. solida*	010010011	0201111121	0021101000
*P. anderssoni*	010001111	0001001021	0011111010
*P. hincksi*	010002001	0102101021	0000111000
*P. sinuosa*	012120111	1221222221	0022111000
*P. birulae*	011010101	0202201111	0101000100
*P. minima*	101002001	1001120010	0010111000
*P. virgula*	100002111	0020001101	1011111000
*P. radeki*	110002111	0000021120	0000111000
*P. perrieri*	101002001	0010001121	1011111000
*S. reticulata*	212000010	0010020011	1000111001

Major features of the tree recovered are its fully pectinate structure, excepting a clade formed by *P*. *perrieri* and *P. virgula*. The five Arctic and North Atlantic species (*P. anderssoni*, *P. hincksi*, *P. birulae*, *P. sinuosa* and *P. solida*) form a clade crownward of the four Atlantic-only species. The Arctic and North Atlantic clade is supported by four synapomorphic characters: bifoliate as opposed to vincularian colonies, relatively elongate autozooids and orifices, and ovicells of small width.

## Discussion

Detailed taxonomical analysis of Arctic and North Atlantic material reveals the existence of at least nine species belonging to the genus *Pseudoflustra*. One of these, previously identified as *Ichthyaria aviculata* Calvet, 1906, was found to be a new species: *P. radeki* sp. nov. Another species, previously assigned to *Smittoidea* as *S. perrieri* (Jullien, 1883), is here transferred to *Pseudoflustra*. Given that *Pseudoflustra*, especially in the North Atlantic, is a deep-water genus which is still poorly sampled, we are aware that further species are likely to be discovered in the future. Busk [51] described *Psileschara maderenis* which may potentially belong to *Pseudoflustra*, yet based on his figures and descriptions, and in the absence of any type material, we are unable to confirm this possibility.

The main characters shared by all nine species of *Pseudoflustra* are the erect colonies supported by cuticular basal rhizoids that allow attachment to soft substrates, frontal shields with marginal areolae but no pseudopores, a cormidial orifice with condyles in the proximolateral corners, the absence of oral spines, large suboral avicularia, and globular hyperstomial ovicells of the smittinoid type. However, as already mentioned, most of these features can also be observed in closely related genera, notably *Smittoidea.*

All species of *Pseudoflustra* are erect; the large quantity of material examined for this study contained no encrusting colonies of *Pseudoflustra*. Examples are known from both bryozoans and other colonial organisms (e.g. hydrozoans, corals) where the dynamics of the environment is apparently able to suppress erect colony growth in favour of current-resistant colonies of low profile [52], [53]. Despite the fact that some colonies of *Pseudoflustra* were collected from shallow-water (5 m depth) environments in the Arctic, where both wave influence and permanent ice impact might be expected to suppress erect growth, no examples were found of encrusting colonies. Also all nine species of *Pseudoflustra* seem to be characteristic of environments where soft sediments dominate. In contrast, the majority of bryozoans depend on firm substrates [54], [55]. The success of *Pseudoflustra* in soft sediment environments is possible because of the rootlets that anchor colonies to the substrate (see Cook [56] for examples of other rooted bryozoans). The fact that *Pseudoflustra* species occur at shallower water-depths in the Arctic than at lower latitudes suggests that the species are strictly thermophobic. In concert with their exclusive adaptation to soft substrates, this argues for an origination of the clade at greater depths.

**Figure 21:**
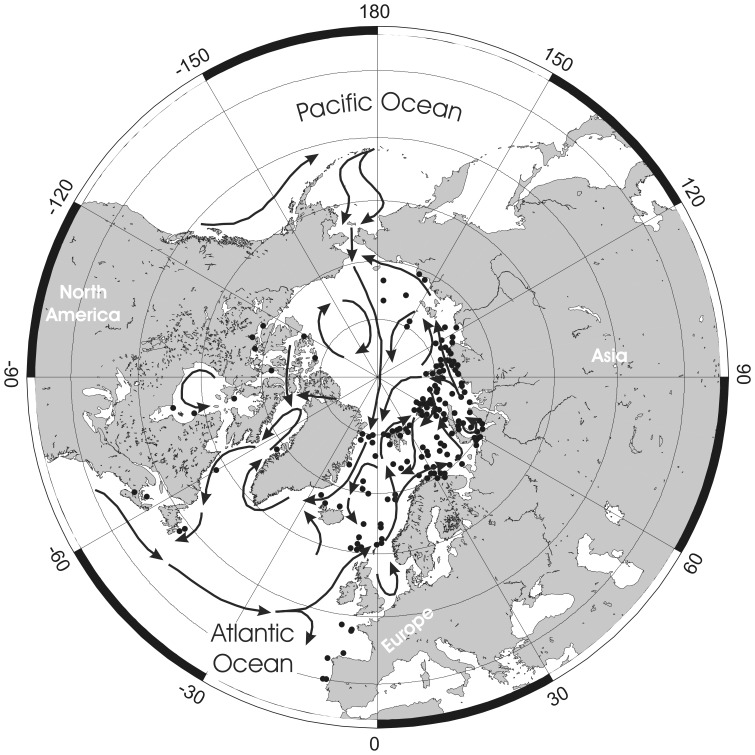
Surface water currents in Arctic Ocean and North Atlantic and distributions of all nine species of *Pseudoflustra*. Note that some symbols represent more than one record as the resolution of the map is insufficient to depict all records.

The very dynamic geology and climate, including warm and cold phases, of the Arctic over the last 65 myrs has clear relevance to the occurrence and distribution of marine biota [1], [3], [5], [8], [57], including *Pseudoflustra*, in the region today. During the Palaeogene the present area of the Arctic was warm and inhabited by thermophilic species, such as crocodiles and turtles [3], [58]. The last 15 myrs have witnessed the onset of glaciation and such important events as the first opening of the Bering Strait (5.4–5.5 Ma) connecting the Arctic with the Pacific Ocean and leading to trans-Arctic biotic interchange between the Pacific and the Atlantic [5], [7]. A number of taxa now inhabiting the Arctic, including algae [2], sea urchins [59], sea stars [60] and molluscs [4], [5], have been shown using both molecular and palaeontological evidence to have their ancestors in the North Pacific. *Pseudoflustra* has no recent or fossil record from the North Pacific or adjacent parts of the Arctic (Bering Southern Chukchi and Beaufort seas) [61]. Among the nine species of *Pseudoflustra,* four (*P. minima*, *P. virgula*, *P. radeki*, *P. perrieri*) have temperate eastern North Atlantic distributions, while the five Arctic-North Atlantic species are most abundant in the Euroasian basin of the Arctic Ocean. Almost all records of *P. anderssoni* and *P. birulai* are from the eastern Arctic. *P. hincksi* and *P. sinuosa* have been recorded in the literature a few times from the western Arctic [13] but these records could not be confirmed here. Only *P. solida* has a broad Arctic distribution. Biogeographical analysis of *Pseudoflustra* species shows distribution in the majority of cases to match surface current patterns in the North Atlantic and Arctic Ocean (Figure 21). In general, the main biotic influence in the western Arctic is from Pacific waters flowing northwards through the Bering Strait, while in the eastern Arctic, where the majority of *Pseudoflustra* species occur, there is a strong influence from the North Atlantic waters [62]. This is not surprising in view of the fact that the volume of Atlantic water entering the Arctic is estimated to be two to five times greater than from the Pacific [63].

Phylogenetic analysis of *Pseudoflustra* places the five Arctic and North Atlantic species in a clade crownward of a paraphyletic sequence of Atlantic-only species (Figure 20). Sister to this Arctic and North Atlantic clade is a clade composed of *P. perrieri* from the Bay of Biscay plus *P. virgula* from the Faroes. This topology suggests that the Arctic and North Atlantic species of *Pseudoflustra* were derived from a deep Atlantic stock. A number of molecular studies have revealed similar phylogeographical patterns [10], [11]. It is difficult to evaluate whether speciation of this clade took place in the Arctic, or outside the Arctic followed by migration into the area after ice retreat and extinction in lower latitudes. Both scenarios are possible and have been shown through population genetic studies of other Arctic biota, including algae, gastropods and crustaceans [64]. It is unlikely that the Arctic and North Atlantic *Pseudoflustra* clade evolved after the last glaciation (18,000 years ago) as this length of time would be insufficient for the amount of speciation necessary [64], [65], [66]. Most Arctic lineage splits are much older than the timing of the Last Glacial Maximum (25–18 ka) [64], [66], [67]. If the Arctic and North Atlantic clade of *Pseudoflustra* evolved in this area before the Last Glacial Maximum it probably vanished as shelf biota were eradicated by glaciations [1]. Species are likely to have tracked cold water by migrating southwards from the Arctic or, alternatively, survived in glacial refugia, small periglacial refugia (isolated northern ice-free areas) or on the continental slope of the Arctic Ocean [64]. Once ice retreated from the shelf at 10–6 ka, *Pseudoflustra* could recolonize the Arctic shelf.

Nesis [68] claimed that some of today’s Arctic shelf faunal elements are ancestors of Arctic deep-sea fauna as faunas of the Atlantic part of the Arctic found refuge in the deep sea. Relatively large numbers of species within Arctic Ocean shared with shelves and continental slope supports that interpretation [69]. The broad depth range of the Arctic and North Atlantic clade of *Pseudoflustra* species is consistent with their survival on the continental slope during the Last Glacial Maximum. However, it must be stressed that the interpretation presented here of the evolutionary history of *Pseudoflustra*, and of the Arctic Ocean colonization in general, is tentative and requires fossil and genetic evidence to be tested.

## Supporting Information

Appendix S1 **Material of** ***Pseudoflustra*** **species examined for this study.**
(DOCX)
