# Supplementary material for: Atlantic Origin of the Arctic Biota? Evidence from Phylogenetic and Biogeographical Analysis of the Cheilostome Bryozoan Genus Pseudoflustra
Source: PLoS One. 2013 Mar 25;8(3):e59152. doi: 10.1371/journal.pone.0059152 (PMC3607580; doi:10.1371/journal.pone.0059152)
Supplement: Appendix S1 — Material of Pseudoflustra species examined for this study. (DOCX) [file pone.0059152.s001.docx]

**Appendix S1.** Material of *Pseudoflustra* species examined for this study.

***Pseudoflustra solida***

**Registered material**

NHM 1911.10.1.1498, Spitsbergen, Norman ex Smitt Collection. NHM 1899.5.1.522, Barents Sea, Hincks Collection. NHM 1899.5.1.523, Barents Sea, 75°10.6’N, 45°19.36’E, Hincks Collection. NHM 1899.5.1.527, Barents Sea, Hincks Collection. NHM 1976.8.6.4, 76°45’N, 32°29’E, depth 128-130 fathoms, Ernest Holt Collection. NHM 1976.8.6.5, 74°02.5’N, 17°36’E, Stn 50, depth 116 fathoms, Ernest Holt Collection. NHM 1934.10.20.86, Kara Sea. NHM 1976.8.4.1, 70°00’N, 28°00’E, depth 180-216 m, 12.05.1975, bottom clay to sandy clay, m/v *Dane*, Arctic Survey, collected by F. H.Thomson. NHM 1976.8.4.2, 71°55’N, 29°04’E, depth 150 fathoms, 29.04.1975, m/v *Dane*, Arctic Survey, collected by F.H. Thomson. NHM 1912.12.21.1014, Varanger Fiord, East Finmark, depth 100-180 fathoms, 1890, Norman Collection. NHM 1912.12.21.1013, Spitsbergen, depth 50 fathoms, Norman ex Smitt Collection. NHM 1963.3.13.31, Spitsbergen, Hinlopen Strait, August 1861, Swedish Expedition. NHM 1891.4.29.9, Kara Sea, Dijmphnae Expedition. NHM 1868.3.13.56, Spitsbergen, Hinlopen Strait, 79°45’N, 20°E, depth 50 fathoms, August 1861, Swedish Expedition, collected by Prof. Loven. NHM 1965.9.4.6, Langenesse Bank, North-West Iceland, depth 80-95 fathoms, 20.08.1953, collected by W.G. Fry. NHM 1976.8.6.3, Arctic, Stn 17, September 1949, collected by E. Holt. NHM 1976.8.6.4, Arctic, Stn 21, collected by E. Holt. NHM 1976.8.6.5, Arctic, Stn 50, May 1950, collected by E. Holt; NHM 2012.3.7.4, Belgica Bank, East Greenland, 79°21.01’N, 07°45.25’W, depth 205-221 m, 12.08.2000, Stn 242, *Polarstern*, cruise ANTXVI/2, Agassiz trawl, collected by B. Bader. NHM 2012.3.7.3, Belgica Bank, East Greenland, 79°19.90’N, 13°35.08’W, depth 164-169 m, 11.08.2000, Stn 228, *Polarstern*, cruise ANTXVI/2, Agassiz trawl, collected by B. Bader. NHM 2012.3.7.6, 78°58,838’N, 11°30,259’E, depth 270-280 m, 31.07.2009, Stn E4, *Oceania*, collected and determined by P. Kuklinski. MM 4163, 77°55’N, 53°16’E, depth 130 fathoms, Jackson–Harmsworth Expedition, Waters Collection. NHMV 16.253, Kara Sea, *Dijmphna* Expedition, Lorenz Collection. NHMV 73.020, Jan Mayen, 1884, Lorenz Collection. CMN 2006-0001, 55°45’N, 82°24’W, 31.08.59, depth 70 m, Powell Collection. CMN 2006-0002, 55°45’N, 82°24’W, 31.08.59, depth 70 m, Powell Collection. CMN 2006-0003, 72°44.1’N, 94°06’W, 25.07.62, depth 35 m, Powell Collection. ZI 1, Barents Sea, 69°25’N, 33°30’E, Stn 59, 26.06.1898, *Murman*, E.N.P.I.M. collection, determined by G.A. Kluge. ZI 2, Barents Sea, 70°3’N, 31°32’E, Stn 25, 17.08.1898, *Murman*, E.N.P.I.M. collection, determined by G.A. Kluge. ZI 3, Barents Sea, 69°3’N, 37°17’E, Stn 106, 31.08.1898, *Murman*, E.N.P.I.M. collection, determined by G.A. Kluge. ZI 4, Barents Sea, 69°27’N, 33°45’E, Stn 5, 24.05.1898, *Pomor*, E.N.P.I.M. collection, determined by G.A. Kluge. ZI 8*,* Barents Sea, 69°37’N, 33°19’E, Stn 71, 23.07.1899, *A. Pervozvanniy,* determined by G.A. Kluge, collected by E.N.P.I.M. ZI 9*,* Barents Sea, 70°34,30’N, 35°10’E, Stn 119, 5.09.1899, *A. Pervozvanniy,* determined by G.A. Kluge, collected by E.N.P.I.M. ZI 10*,* Barents Sea, 69°9’N, 37°32’E, Stn 28, 15.06.1899, *A. Pervozvanniy,* determined by G.A. Kluge, collected by E.N.P.I.M. ZI 11*,* Barents Sea, 69°39’N, 41°48’E, Stn 76, 23.07.1899, *A. Pervozvanniy,* determined by G.A. Kluge, collected by E.N.P.I.M. ZI 12*,* Barents Sea, 69°13,30’N, 39°30’E, Stn 73, 22.07.1899, *A. Pervozvanniy,* determined by G.A. Kluge, collected by E.N.P.I.M. ZI 13*,* Barents Sea, 71°33’N, 32°06’E, Stn 40, 24.06.1899, *A. Pervozvanniy,* determined by G.A. Kluge, collected by E.N.P.I.M. ZI 14*,* Barents Sea, 72°13,30’N, 32°10’E, Stn 41, 25.06.1899, *A. Pervozvanniy,* determined by G.A. Kluge, collected by E.N.P.I.M. ZI 15*,* Barents Sea, 69°40’N, 35°15’E, Stn 12, 24.06.1899, *A. Pervozvanniy,* determined by G.A. Kluge, collected by E.N.P.I.M. ZI 16*,* Barents Sea, 75°00’N, 31°10’E, Stn 101, 24.08.1899, *A. Pervozvanniy,* determined by G.A. Kluge, collected by E.N.P.I.M. ZI 17*,* Barents Sea, 70°00’N, 35°05’E, Stn 118, 24.08.1899, *A. Pervozvanniy,* determined by G.A. Kluge, collected by E.N.P.I.M. ZI 18*,* Barents Sea, 73°38’N, 27°14’E, Stn 106, 27.08.1899, *A. Pervozvanniy,* determined by G.A. Kluge, collected by E.N.P.I.M. ZI 19*,* Barents Sea, 69°34,30’N, 33°31’E, Stn 57, 13.07.1899, *A. Pervozvanniy,* determined by G.A. Kluge, collected by E.N.P.I.M. ZI 20*,* Barents Sea, 69°45,30’N, 36°07’E, Stn 242, 20.06.1900, *A. Pervozvanniy,* determined by G.A. Kluge, collected by E.N.P.I.M. ZI 22*,* Barents Sea, 70°55’N, 33°30’E, Stn 204, 29.05.1900, *A. Pervozvanniy,* determined by G.A. Kluge, collected by E.N.P.I.M. ZI 23*,* Barents Sea, 69°31’N, 33°17’E, Stn 266, 24.07.1900, *A. Pervozvanniy,* determined by G.A. Kluge, collected by E.N.P.I.M. ZI 24*,* Barents Sea, 73°25’N, 46°48’E, Stn 253, 27.06.1900, *A. Pervozvanniy,* determined by G.A. Kluge, collected by E.N.P.I.M. ZI 25*,* Barents Sea, 69°22’N, 32°56’E, Stn 312, 23.08.1900, *A. Pervozvanniy,* determined by G.A. Kluge, collected by E.N.P.I.M. ZI 26*,* Barents Sea, 71°35’N, 33°08’E, Stn 260, 14.07.1900, *A. Pervozvanniy,* determined by G.A. Kluge, collected by E.N.P.I.M. ZI 27*,* Barents Sea, 73°25’N, 46°480’E, Stn 253, 30.06.1900, *A. Pervozvanniy,* determined by G.A. Kluge, collected by E.N.P.I.M. ZI 28*,* Barents Sea, 73°08’N, 33°30’E, Stn 258, 30.06.1900, *A. Pervozvanniy,* determined by G.A. Kluge, collected by E.N.P.I.M. ZI 29*,* Barents Sea, 70°23’N, 34°06’E, Stn 469, 24.06.1901, *A. Pervozvanniy,* determined by G.A. Kluge, collected by E.N.P.I.M. ZI 30*,* Barents Sea, 70°30’N, 31°59’E, Stn 467, 24.06.1901, *A. Pervozvanniy,* determined by G.A. Kluge, collected by E.N.P.I.M. ZI 31*,* Barents Sea, 72°02.30’N, 46°00’E, Stn 617, 24.08.1901, *A. Pervozvanniy,* determined by G.A. Kluge, collected by E.N.P.I.M. ZI 32*,* Barents Sea, 71°17’N, 32°57’E, Stn 463, 22.06.1901, *A. Pervozvanniy,* determined by G.A. Kluge, collected by E.N.P.I.M. ZI 33*,* Barents Sea, 69°23’N, 32°55’E, Stn 429, 26.05.1901, *A. Pervozvanniy,* determined by G.A. Kluge, collected by E.N.P.I.M. ZI 34*,* Barents Sea, 73°40’N, 40°20’E, Stn 516, 16.07.1901, *A. Pervozvanniy,* determined by G.A. Kluge, collected by E.N.P.I.M. ZI 35*,* Barents Sea, 69°27.30’N, 34°41’E, Stn 474, 25.06.1901, *A. Pervozvanniy,* determined by G.A. Kluge, collected by E.N.P.I.M. ZI 36*,* Barents Sea, 69°44.30’N, 30°47’E, Stn 882, 4.08.1903, *A. Pervozvanniy,* determined by G.A. Kluge, collected by E.N.P.I.M. ZI 37*,* Barents Sea, 70°30’N, 36°27’E, Stn 913, 27.08.1903, *A. Pervozvanniy,* determined by G.A. Kluge, collected by E.N.P.I.M. ZI 38*,* Barents Sea, 70°04’N, 39°45’E, Stn 883, 4.08.1903, *A. Pervozvanniy,* determined by G.A. Kluge, collected by E.N.P.I.M. ZI 39*,* Barents Sea, 72°30’N, 43°42’E, Stn 1360, 14.08.1906, *A. Pervozvanniy,* determined by G.A. Kluge, collected by E.N.P.I.M. ZI 40*,* Barents Sea, 74°00’N, 45°25’E, Stn 44, 24.06.1901, *Ermak,* determined by G.A. Kluge, collected by G.I. Chernyshov. ZI 41*,* Barents Sea, 79°38’N, 50°38’E, Stn 65, 28.07.1901, *Ermak,* determined by G.A. Kluge, collected by G.I. Chernyshov. ZI 42*,* Barents Sea, 78°07’N, 63°33’E, Stn 85, 4.08.1901, *Ermak,* determined by G.A. Kluge, collected by G.I. Chernyshov. ZI 43*,* Kara Sea, 80°26’N, 64°14’E, Stn 82, 3.08.1901, *Ermak,* determined by G.A. Kluge, collected by G.I. Chernyshov. ZI 44*,* Kara Sea, 79°45’N, 65°09’E, Stn 83, 3.08.1901, *Ermak,* determined by G.A. Kluge, collected by G.I. Chernyshov. ZI 45*,* Barents Sea, 74°31’N, 53°20’E, Stn 48, 26.06.1901, *Ermak,* determined by G.A. Kluge, collected by G.I. Chernyshov. ZI 46*,* Barents Sea, 78°00’N, 52°57’E, Stn 61, 26.07.1901, *Ermak,* determined by G.A. Kluge, collected by G.I. Chernyshov. ZI 47*,* Barents Sea, 77°53’N, 61°29’E, Stn 76, 1.08.1901, *Ermak,* determined by G.A. Kluge, collected by G.I. Chernyshov. ZI 48*,* Barents Sea, 74°32’N, 54°20’E, Stn 48, 2.07.1901, *Ermak,* determined by G.A. Kluge, collected by G.I. Chernyshov. ZI 49*,* Barents Sea, Kola Bay, Stn 3, 7.08.1908, *A. Kovalevskiy,* determined by G.A. Kluge, collected by K.M. Deriugin. ZI 51, Kola Bay, depth 62 m, *Zaria*, trawl, determined by G.A. Kluge. ZI 56*,* Barents Sea, 70°45’N, 32°57’E, Stn 261, 15.07.1900, *A. Pervosvanniy,* determined by G.A. Kluge, collected by E.N.P.I.M. ZI 57, 69°39’N, 46°16’E, Stn 16, 20.07.1900, depth 85 m, *A. Kovalevsky*, determined by G.A, Kluge. ZI 58, 69°37’N, 56°43’E, Stn 3, 27.07.1900, depth 30 m, *Zaria*, trawl, determined by G.A. Kluge. ZI 59, 76°08’N, 93°30’E, Stn 26, 22.09.1900, depth 28-32 m, *Zaria*, trawl, determined by G.A. Kluge. ZI 60, 76°08’N, 93°30’E, Stn 25, 22.09.1900, depth 30 m, *Zaria*, trawl, determined by G.A. Kluge. ZI 61, 76°08’N, 95°06.5’E, Stn 27, 22.09.1900, depth 19-20 m, *Zaria*, trawl, determined by G.A, Kluge. ZI 62, 76°08’N, 95°06.1’E, Stn 33, 16.07.1900, depth 18-20 m, *Zaria*, trawl, determined by G.A. Kluge. ZI 63, Taymyrskiy Bay, 76°59.1’N, 100°19.5’E, Stn 44, 31.08.1900, depth 28 m, *Zaria*, trawl, determined by G.A. Kluge. ZI 64, Laptev Sea, 75°32.5’N, 118°32’E, Stn 48, 05.09.1900, depth 30 m, *Zaria*, trawl, determined by G.A. Kluge. ZI 65, Laptev Sea, 77°01’N, 114°35’E, Stn 46, 03.09.1900, depth 60 m, *Zaria*, trawl, determined by G.A, Kluge. ZI 66, near Nansen Island (Kara Sea), Stn 43, 29.08.1901, *Zaria*, trawl, determined by G.A. Kluge. ZI 67, near Dikson Island (Kara Sea), 76°20’N, 83°50’E, Stn 11, 12.08.1933, *Sibiriakov*, trawl, determined by G.A. Kluge, collected by G.I. Gorbunov. ZI 68, Laptev Sea, 77°16’N, 110°52’E, Stn 14, 29.08.1932, *Sibiriakov*, trawl, determined by G.A. Kluge, collected by G.I. Gorbunov. ZI 70, Laptev Sea, 76°08’N, 115°50’E, Stn 34, 14.09.1933, *Sibiriakov*, trawl, determined by G.A. Kluge, collected by G.I. Gorbunov. ZI 71, Laptev Sea, 76°50’N, 113°13’E, Stn 15, 23.08.1932, *Sibiriakov*, trawl, determined by G.A. Kluge, collected by G.I. Gorbunov. ZI 74, Kara Sea, 76°59’N, 94°58’E, Stn 40, 27.09.1933, *Sibiriakov*, trawl, determined by G.A. Kluge, collected by G.I. Gorbunov. ZI 75, Kara Sea, 76°14’N, 83°38’E, Stn 10, 2.08.1933, *Sibiriakov*, trawl, determined by G.A. Kluge, collected by G.I. Gorbunov.

ZI 76, Kara Sea, 75°47’N, 84°36’E, Stn 6, 09.08.1933, *Sibiriakov*, trawl, determined by G.A. Kluge, collected by G.I. Gorbunov. ZI 80, Kara Sea, 77°13’N, 96°04’E, Stn 39, 25.09.1933, *Sibiriakov*, trawl, determined by G.A. Kluge, collected by G.I. Gorbunov. ZI 81, Wilkitskiy Straight, 78°02.5’N, 105°24’E, Stn 22, 05.09.1932, *Pusanov*, determined by G.A. Kluge, collected by V.L.Bagin and N.N. Kondakov. ZI 82, Wilkitskiy Straight, 78°11'N, 105°38'E, 05.09.1932*, Rusanov,* Stn 23, collected by N.N. Kondakov, V.L. Vagin, determined by G.A. Kluge. ZI 83, Kara Sea, 70°46'N, 58°28'E, 14.07.1931, *Rusanov,* stn 19, collected by G.I Gorbunov, determined by G.A. Kluge. ZI 84, between Spitsbergen and FJL, 80°09.9'N, 33°59,5'E, 16.08.1935, *Sadko,* stn 24/55, collected by G.I. Gorbunov, determined by G.A. Kluge. ZI 85, Kara Sea, 70°50'N, 57°34'E, 13.08.1931, *Rusanov,* stn 17, collected by G.I Gorbunov, determined by G.A. Kluge. ZI 89, Shokalskiy Straight, 79°15.5'N, 100°04'E, 20.08.1932, *Rusanov,* stn 18, collected by V.L. Vagin, determined by G.A. Kluge. ZI 92, Kara Sea, 71°55'N, 66°02'E, 9.08.1931, *Rusanov*, stn 11, collected by G.I. Gorbunov, determined by G.A. Kluge. ZI 92, Kara Sea, 69°53'N, 64°01'E, 20.08.1931, *Rusanov*, stn 22, collected by G.I. Gorbunov, determined by G.A. Kluge. ZI 94, Kara Sea, 73°39'N, 62°37'E, 19.09.1931, *Rusanov*, stn 41, collected by G.I. Gorbunov, determined by G.A. Kluge. ZI 95, Kara Sea, 74°52'N, 75°15'E, 8.08.1931, *Rusanov*, stn 8, collected by G.I. Gorbunov, determined by G.A. Kluge. ZI 96, Kara Sea, 70°23'N, 64°00'E, 20.08.1931, *Rusanov*, stn 21, collected by G.I. Gorbunov, determined by G.A. Kluge. ZI 101, Kara Sea, 79°09'N, 78°30'E, 18.08.1930, *Sedov*, stn 48/18, collected by G.I. Gorbunov, determined by G.A. Kluge. ZI 103, Kara Sea, 78°02'N, 86°30'E, 2.09.1930, *Sedov*, stn 62/32, collected by G.I. Gorbunov, determined by G.A. Kluge. ZI 106, Kara Sea, 80°26'N, 88°57'E, 31.08.1930, *Sedov*, stn 58/28, collected by G.I. Gorbunov, determined by G.A. Kluge. ZI 108, Kara Sea, 78°33'N, 86°20'E, 2.09.1930, *Sedov*, stn 61/31, collected by G.I. Gorbunov, determined by G.A. Kluge. ZI 116, Barents Sea, 78°40'N, 58°00'E, 4.09.1929, *Sedov*, stn 24, collected by G.I. Gorbunov, determined by G.A. Kluge. ZI 120, Franz Josef Land, 80°20'N, 58°48'E, 26.07.1930, *Sedov*, stn 36, collected by G.I. Gorbunov, determined by G.A. Kluge. ZI 167/1740, Polar Basin, 79°30'N, 150°10'E, 12.04.1938, *Sadko*, stn 93, collected by G.I. Gorbunov, determined by G.A. Kluge. ZI 171/2535, Laptev Sea, 77°10.3’N, 120°50'E, 19.08.1937, *Sadko*, stn 14, collected by G.I. Gorbunov, determined by G.A. Kluge. ZI 176/3056, Polar Basin, 80°57'N, 20°51'E, 17.08.1899, *Ermak*, stn 27, collected by A.G. Chernyshov, determined by G.A. Kluge. ZI 177/3057, Polar Basin, 81°1'N, 19°28'E, 18.08.1899, *Ermak*, stn 28, collected by A.G. Chernyshov, determined by G.A. Kluge. ZI 178/5425, Polar Basin, 80°58'N, 17°17'E, 10.10.1955, *Litke*, stn 46, collected by V.M. Koltun, determined by M.G. Gostilovskaya. ZI 179/5436, Polar Basin, 82°11'N, 60°57'E, 15.09.1955, *Litke*, stn 13, collected by V.M.Koltun, determined by M.G. Gostilovskaya. ZI 181/5526, Polar Basin, 81°57'N, 50°16'E, 16.09.1955, *Litke*, stn 17, collected by V.M. Koltun, determined by M.G. Gostilovskaya. ZI 182/5527, Polar Basin, 81°52'N, 60°32'E, 15.09.1955, *Litke*, stn 14, collected by V.M. Koltun, determined by M.G. Gostilovskaya. ZI 186/13328, Greenland Sea, 80°22,9'N, 9°39'W, 26.08.1956, *Ob’*, stn 26, collected by V.M. Koltun, determined by M.G. Gostilovskaya. ZI 187/13411, Greenland Sea, 78°00'N, 11°36'W, 20.08.1958, *Lena*, stn 22, collected by M. Petrovskaya, determined by M.G. Gostilovskaya. ZI 191/2942, Kara Sea, Stn 61/102, *Sadko*, 14.09.1935, determined by G.A. Kluge, collected by G.I. Gorbunov.

### **Unregistered material**

Faroe Islands, 63°03.18’N, 7°41.58’W, depth 804 m, 16.05.1988, Stn 275, BIOFAR project, detritus sledge, determined P.J. Hayward. Faroe Islands, 61°00.16’N, 5°13.89’W, depth 604 m, 22.06.1989, Stn 481, BIOFAR project, detritus sledge, determined P.J. Hayward. Faroe Islands, 60°58.51’N, 9°55.84’W, depth 1083 m, 23.07.1989, Stn 490, BIOFAR project, detritus sledge, determined P.J. Hayward. Faroe Islands, 61°17.06’N, 10°32.07’W, depth 1157 m, 30.09.1990, Stn 736, BIOFAR project, detritus sledge, determined P.J. Hayward.

***Pseudoflustra hincksi***

**Registered material**

### NHM 1965.9.4.5, Langenesse Bank, North-West Iceland, depth 80-95 fathoms, 20.08.1953, collected by W.G. Fry. ZI 1, Barents Sea, trawl no. 37, 1898, Pomor, E.N.P.I.M. collection, determined by G.A. Kluge. ZI 2, Barents Sea, dredge no 59, 08.08.1898, Murman, E.N.P.I.M. collection, determined by G.A. Kluge. ZI 3, Barents Sea, Stn 166, 26.03.1900, A. Pervozvanniy, E.N.P.I.M. collection, determined by G.A. Kluge. ZI 4, Barents Sea, Stn 467, 11.06.1901, A. Pervozvanniy, E.N.P.I.M. collection, determined by G.A. Kluge. ZI 5, Barents Sea, dredge 15, 2.06.1893, E.N.P.I.M. collection, determined by G.A. Kluge. ZI 7, Barents Sea, Stn 86, 4.08.1901, Ermak, collected by A.G. Chernyshev, determined by G.A. Kluge. ZI 8, Kara Sea, 73°45’N, 68°07’E, Stn 39, Rusanov, trawl, 18.09.1931, determined by G.A. Kluge, collected by G.P. Gorbunov. ZI 9, Shokalskiy Straight, 79°15.5’N, 100°04’E, Stn 18, Rusanov, 20.08.1932, determined by G.A. Kluge, collected by V.L.Vagin. ZI 10, Kara Sea, 80°26’N, 88°57’E, Stn 58, Sedov, 18.08.1932, determined by G.A. Kluge, collected by G.P. Gorbunov. ZI 11, Franz Josef Land, 80°51’N, 52°00’E, Stn 22, Sedov, 25.08.1929, determined by G.A. Kluge, collected by G.P. Gorbunov. ZI 12, Wilkitzki Strait, 77°56’N, 103°33’E, Stn 26, Rusanov, trawl, 6-7.09.1932, determined by G.A. Kluge, ZI 13, 80°20’N, 58°47’E, Stn 36, depth 5 m, 26.07.1930, Sedov, collected by G.P. Gorbunov, determined by G.A. Kluge. ZI 14/567, 78°17’N, 104°52’E, Stn 175, depth 44.5 m, 09.09.1948, Litke, collected by V.M. Koltun, V.L. Vagin, determined by M.G. Petrovskaya. ZI 15/568, 80°14’N, 80°12’E, Stn 189A, depth 64 m, 02.10.1948, Litke, collected by V.M. Koltun, V.L. Vagin, determined by M.G. Petrovskaya. ZI 17/570, 79°54.5’N, 103°33’E, Stn 132, depth 348 m, 26.07.1948, Litke, collected by V.M. Koltun, V.L. Vagin, determined by M.G. Petrovskaya. ZI 19/572, 79°01’N, 69°56’E, Stn 83, depth 509 m, 12.08.1948, Litke, collected by V.M. Koltun, V.L. Vagin, determined by M.G. Petrovskaya. ZI 20/573, 78°34’N, 99°33’E, Stn 172, depth 132 m, 26.08.1948, Litke, collected by V.M. Koltun, V.L. Vagin, determined by M.G. Petrovskaya. ZI 21/574, 77°33’N, 106°05’E, Stn 153, depth 70 m, 03.09.1948, Litke, collected by V.M. Koltun, V.L. Vagin, determined by M.G. Petrovskaya.ZI 22/575, 78°28’N, 114°36’E, Stn 145, depth 459 m, 01.09.1948, Litke, collected by V.M. Koltun, V.L. Vagin, determined by M.G. Petrovskaya. ZI 23/576, 79°26’N, 107°48’E, Stn 127, depth 1073 m, 30.08.1948, Litke, collected by V.M. Koltun, V.L. Vagin, determined by M.G. Petrovskaya. ZI 24/577, 78°28’N, 72°12’E, Stn 87, depth 461 m, 13.08.1948, Litke, collected by V.M. Koltun, V.L. Vagin, determined by M.G. Petrovskaya. ZI 26/579, 80°30’N, 90°03’E, Stn 183, depth 174 m, 29.09.1948, Litke, collected by V.M. Koltun, V.L. Vagin, determined by M.G. Petrovskaya. ZI 27/1764, Kara Sea, Stn 65/107, Sadko, trawl, 17.09.1935, determined by G.A. Kluge, collected by G.I. Gorbunov. ZI 28/3324, Barents Sea, Stn 29, Diana, 26.04.1953, collected and determined by M.G. Gostilovskaya. ZI 29/4992, Polar Basin, 76°47’N, 173°17’E, Stn 2, SP-4, 19-20.06.1954, determined by G.A. Kluge, collected by A.A.R.I. ZI 30/5429, Arctic Ocean near Franz Joseph Land, Stn 13, 15.09.1955, Litke, determined by M.G. Gostilovskaya, collected by V.M. Koltun. ZI 31/5522, Arctic Ocean near Franz Joseph Land, Stn 17, 16.09.1955, Litke, collected by V.M. Koltun, determined by M.G. Gostilovskaya. ZI 33/5528, Franz Josef Land, Stn 14, Litke, 15.09.1955, determined by M.G. Gostilovskaya, collected by V.M. Koltun. ZI 34/50535, 64°44.5438’N, 04°47.9748’E, Stn 271-01 ASPI-7, depth 798 m, 22.05.2006, Pourquoi Pas, collected in frames of HERMES program, cruise leader H. Nouze, determined by N.V. Denisenko. ZI 35/50536, 64°39,97’N, 5°44.23’E, Stn 272-02, GBT1, R–11, depth 738 m, 26.05.2006, Pourquoi Pas, collected in frame of HERMES program, cruise leader H. Nouze, determined by N.V. Denisenko. ZI 36/50537, 64°45.2776’N, 05°05.6080’E, Stn 271-01 Pruer-2, depth 718 m, 22.05.2006, collected in frame of HERMES program, cruise leader H. Nouze, determined by N.V. Denisenko. ZI 37/50538, 64°39.9976’N, 05°17.347’E, Stn 272-02 GTB-8, depth 736 m, 26.05.2006, Pourquoi Pas, collected in frame of HERMES program, cruise leader H. Nouze, determined by N.V. Denisenko.

### **Unregistered material**

### Faroe Islands, 62°37.68’N, 4°40.37’W, depth 683 m, 17.07.1987, Stn 15, BIOFAR project, Rothlisberg & Pearcy epibenthic sampler, determined P.J. Hayward. Faroe Islands, 62°25.45’N, 3°31.60’W, depth 601 m, 08.05.1988, Stn 171, BIOFAR project, Rothlisberg & Pearcy epibenthic sampler, determined P.J. Hayward. Faroe Islands, 62°19.12’N, 3°54.79’W, depth 507 m, 09.05.1988, Stn 172, BIOFAR project, Rothlisberg & Pearcy epibentic sampler, determined P.J. Hayward. Faroe Islands, 61°08.96’N, 4°54.22’W, depth 703 m, 12.05.1988, Stn 230, BIOFAR project, detritus sledge, determined P.J. Hayward. Faroe Islands, 63°00.79’N, 7°49.22’W, depth 698 m, 16.05.1988, Stn 274, BIOFAR project, detritus sledge, determined P.J. Hayward. Faroe Islands, 63°03.18’N, 7°41.58’W, depth 804 m, 16.05.1988, Stn 275, BIOFAR project, detritus sledge, determined P.J. Hayward. Faroe Islands, 62°50.24’N, 9°34.42’W, depth 509 m, 02.06.1989, Stn 424, BIOFAR project, Rothlisberg & Pearcy epibenthic sampler, determined P.J. Hayward. Faroe Islands, 62°54.92’N, 7°00.23’W, depth 675 m, 04.06.1989, Stn 458, BIOFAR project, detritus sledge, determined P.J. Hayward. Faroe Islands, 62°59.42’N, 6°57.52’W, depth 910 m, 04.06.1989, Stn 459, BIOFAR project, detritus sledge, determined P.J. Hayward. Faroe Islands, 61°03.66’N, 4°43.92’W, depth 973 m, 21.07.1989, Stn 478, BIOFAR project, detritus sledge, determined P.J. Hayward. Faroe Islands, 60°26.87’N, 8°22.64’W, depth 714 m, 25.07.1989, Stn 500, BIOFAR project, detritus sledge, determined P.J. Hayward. Faroe Islands, 60°30.20’N, 8°15.70’W, depth 804 m, 25.07.1989, Stn 501, BIOFAR project, detritus sledge, determined P.J. Hayward. Faroe Islands, 60°38.38’N, 7°31.02’W, depth 1038 m, 15.07.1990, Stn 705, BIOFAR project, detritus sledge, determined P.J. Hayward. Faroe Islands, 61°09.90’N, 5°01.80’W, depth 610 m, 28.09.1990, Stn 719, BIOFAR project, detritus sledge, determined P.J. Hayward. Faroe Islands, 60°32.20’N, 7°08.20’W, depth 949 m, 29.09.1990, Stn 730, BIOFAR project, detritus sledge, determined P.J. Hayward. Faroe Islands, 60°29.70’N, 7°14.10’W, depth 1042 m, 29.09.1990, Stn 731, BIOFAR project, detritus sledge, determined P.J. Hayward. Faroe Islands, 62°46.80’N, 5°51.50’W, depth 497 m, 03.10.1990, Stn 749, BIOFAR project, detritus sledge, determined P.J. Hayward. Faroe Islands, 62°48.70’N, 5°44.10’W, depth 600 m, 03.10.1990, Stn 750, BIOFAR project, detritus sledge, determined P.J. Hayward. Faroe Islands, 62°49.80’N, 5°35.00’W, depth 708 m, 03.10.1990, Stn 751, BIOFAR project, detritus sledge, determined P.J. Hayward. Faroe Islands, 62°19.90’N, 3°06.00’W, depth 645 m, 06.10.1990, Stn 769, BIOFAR project, detritus sledge, determined P.J. Hayward. Faroe Islands, 62°20.40’N, 3°11.70’W, depth 583 m, 06.10.1990, Stn 770, BIOFAR project, detritus sledge, determined P.J. Hayward. Faroe Islands, 62°24.70’N, 3°52.20’W, depth 560 m, 08.10.1990, Stn 774, BIOFAR project, detritus sledge, determined P.J. Hayward.

***Pseudoflustra anderssoni***

**Registered material**

### NHM 2012.3.7.2, Belgica Bank, East Greenland, 78°59.89’N, 11°13.02’W, depth 108-113 m, 10.08.2000, Stn 215, Polarstern, cruise ANTXVI/2, Agassiz trawl, collected by B. Bader. ZI 1, Barents Sea, Stn 237, 15.06.1900, A. Pervozvanniy, determined by G.A. Kluge, collected by E.N.P.I.M. ZI 2, Barents Sea, Stn 106, 15.06.1900, A. Pervozvanniy, determined by G.A. Kluge, collected E.N.P.I.M. ZI 3, Barents Sea, Stn 261, 15.07.1900, A. Pervozvanniy, determined by G.A. Kluge, collected by E.N.P.I.M. ZI 4, Barents Sea, Stn 85, 4.08.1901, Ermak, determined by G.A. Kluge, collected by A.G. Chernyshov. ZI 5, Barents Sea, 79°15’N, 60°44’E, 2.08.1901, Ermak, determined by G.A. Kluge, collected by A.G. Chernyshov. ZI 6, Kara Sea, 73°45’N, 68°07’E, Stn 39, Rusanov, trawl, 18.09.1931, determined by G.A. Kluge, collected by G.P. Gorbunov. ZI 7, Shokal’skiy Strait (Laptev Sea), 78°49’N, 100°10’E, Stn 11, Rusanov, 19.08.1932, collected by N.N. Kondakov and V.L. Vagin, determined by G.A. Kluge. ZI 8, Kara Sea, 73°39’N, 62°37’E, Stn 11, Rusanov, trawl, 19.09.1931, determined by G.A. Kluge, collected by G.P. Gorbunov. ZI 9, Kara Sea, 79°55’N, 88°58’E, Stn 57, Sedov, 31.08.1930, determined by G.A. Kluge, collected by G.P. Gorbunov. ZI 10, Wilkitzki Strait, 77°56’N, 103°33’E, Stn 26, Rusanov, trawl, 6-7.09.1932, determined by G.A. Kluge, collected by V.L. Vagin. ZI 11, Kara Sea, 77°16’N, 94°56’E, Stn 38, Sibiriakov, 22.09.1933, determined by G.A. Kluge, collected by G.P. Gorbunov. ZI 12, Kara Sea, 70°50’N, 57°34’E, Stn 17, Rusanov, trawl, 13.08.1931, determined by G.A. Kluge, collected by G.P. Gorbunov. ZI 13/2456, Polar Basin, 79°30’N, 150°10’E, Stn 93, Sadko, 12.04.1938, determined by G.A. Kluge, collected by G.P. Gorbunov. ZI 14/2858, between Spitsbergen and Franz Josef Land, Stn 24/55, Sadko, 10.08.1935, determined by G.A. Kluge, collected by G.P. Gorbunov. ZI 15/5428, Spitsbergen, 80°57’N, 50°16’E, Stn 46, Litke, 10.10.1955, collected by V.M Koltun, determined by M.G. Gostilovskaya. ZI 16/5519, Polar Basin, Franz Josef Land, 81°57’N, 50°16’E, Stn 17, Litke, 16.09.1955, collected by V.M Koltun, determined by M.G. Gostilovskaya.

**Unregistered material**

### Faroe Islands, 62°50.82’N, 6°39.70’W, depth 509 m, 10.05.1988, Stn 189, BIOFAR project, detritus sledge, determined P.J. Hayward. Faroe Islands, 61°08.96’N, 4°54.22’W, depth 703 m, 12.05.1988, Stn 230, BIOFAR project, detritus sledge, determined P.J. Hayward. Faroe Islands, 61°03.66’N, 4°43.92’W, depth 973 m, 21.07.1989, Stn 478, BIOFAR project, detritus sledge, determined P.J. Hayward.

***Pseudoflustra sinuosa***

**Registered material**

### NHM 1911.10.1.1498A, Varanger Fjord, East Finmark, 100–150 fathoms, 1890, Norman Collection. NHM 2012.3.7.5, Belgica Bank, East Greenland, 79°21.01’N, 07°45.25’W, depth 205-221 m, 12.08.2000, Polarstern, stn 242, cruise ANTXVI/2, Agassiz trawl, collected by B. Bader. ZI 1, Barents Sea, 69°55'N, 30°39’15’’E, Stn 303, 15.06.1900, A. Pervozvanniy, determined by G.A. Kluge, collected by E.N.P.I.M. ZI 2, Barents Sea, 70°23'N, 34°06’E, Stn 469, 24.06.1900, A. Pervozvanniy, determined by G.A. Kluge, collected by E.N.P.I.M. ZI 3, Barents Sea, 71°35'N, 33°08’E, Stn 260, 14.06.1900, A. Pervozvanniy, determined by G.A. Kluge, collected by E.N.P.I.M. ZI 4, Barents Sea, 73°38'N, 27°14'E, Stn 106, 15.06.1900, A. Pervozvanniy, determined by G.A. Kluge, collected by E.N.P.I.M. ZI 5, Barents Sea, 70°3'N, 31°32’E, Stn 25, 17.06.1900, A. Murman, determined by G.A. Kluge, collected by E.N.P.I.M. ZI 6, 77°53'N, 61°29'E, 01.08.1901, Ermak, Stn 76, collected by A.G. Chernyshev, determined by G.A. Kluge. ZI 7, 78°07'N, 63°33'E, 04.08.1901, Ermak, stn 85, collected by A.G. Chernyshev, determined by G.A. Kluge. ZI 8, 79°15'N, 60°44'E, depth 323 m, 02.08.1901, Ermak, stn 80, collected by A.G. Chernyshov, determined by G.A. Kluge. ZI 9, Kara Sea, 76°50'N, 93°46'E, 28.08.1933, Sibiriakov, stn 25, collected by G.I. Gorbunov, determined by G.A. Kluge. ZI 10, Kara Sea, 77°11'N, 96°15'E, 28.08.1933, Sibiriakov, stn 26, collected by G.I. Gorbunov, determined by G.A. Kluge. ZI 11, Wilkitskiy Straight, 78°2.5'N, 105°24'E, 05.09.1932, Rusanov, stn 22, collected by N.N. Kondakov, V.L. Vagin, determined by G.A. Kluge. ZI 12, 70°46'N, 58°28'E, depth 180 m, 14.08.1931, Rusanov, stn 19, collected by N.N. Kondakov, V.L. Vagin, determined by G.A. Kluge. ZI 13, 70°50'N, 57°34'E, 13.08.1931, Rusanov, stn 17, collected by N.N. Kondakov, V.L. Vagin, determined by G.A. Kluge. ZI 15, Wilkitskiy Straight, 77°46.5'N, 100°19'E, depth 100 m, 08.09.1932, Rusanov, stn 32, collected by N.N. Kondakov, V.L. Vagin, determined by G.A. Kluge. ZI 16, Kara Sea, 71°26'N, 57°34'E, 13.09.1931, Rusanov, stn 32, collected by G.I. Gorbunov, determined by G.A. Kluge. ZI 15, Shokalskiy Straight, 78°49'N, 100°10'E, depth 276 m, 19.08.1932, Rusanov, stn 11, collected by N.N. Kondakov, V.L. Vagin, determined by G.A. Kluge. ZI 18, Kara Sea, 71°55'N, 66°02'E, 9.08.1931, Rusanov, stn 11, collected by G.I. Gorbunov, determined by G.A. Kluge. ZI 19, Kara Sea, 73°39'N, 62°37'E, 19.09.1931, Rusanov, stn 41, collected by G.I. Gorbunov, determined by G.A. Kluge. ZI 21, Kara Sea, 79°09'N, 78°30'E, 18.08.1930, Sedov, Stn 48, collected by G.I. Gorbunov, determined by G.A. Kluge. ZI 22, Kara Sea, 78°02'N, 86°30'E, 2.09.1930, Sedov, Stn 62(32), collected by G.I. Gorbunov, determined by G.A. Kluge. ZI 23, Kara Sea, 79°55'N, 88°58'E, 31.08.1930, Sedov, Stn 57(23), collected by G.I. Gorbunov, determined by G.A. Kluge. ZI 24, Kara Sea, 80°26'N, 88°57'E, 31.08.1930, Sedov, Stn 58(28), collected by G.I. Gorbunov, determined by G.A. Kluge. ZI 25, Kara Sea, 78°33'N, 86°20'E, 2.09.1930, Sedov, Stn 61(31), collected by G.I. Gorbunov, determined by G.A. Kluge. ZI 26, Kara Sea, 79°10'N, 78°50'E, 17.08.1930, Sedov, Stn 47(17), collected by G.I. Gorbunov, determined by G.A. Kluge. ZI 27, Severnaya Semlia archipelago, 77°33'N, 79°45'E, 20.08.1930, Sedov, Stn 51(21), collected by G.I. Gorbunov, determined by G.A. Kluge. ZI 28, Kara Sea, 78°58'N, 68°25'E, 12.08.1930, Sedov, Stn 44(14), collected by G.I. Gorbunov, determined by G.A. Kluge. ZI 29, Barents Sea, 78°40'N, 58°00'E, 4.09.1930, Sedov, Stn 24, collected by G.I. Gorbunov, determined by G.A. Kluge. ZI 30, Franz Josef Land archipelago, 80°51'N, 52°00'E, 25.08.1930, Sedov, Stn 22, collected by G.I. Gorbunov, determined by G.A. Kluge. ZI 31, Wilkitskiy Strait, 77°56'N, 103°33'E, 6–7.09.1932, Rusanov, Stn 26, collected by V.L. Vagin, determined by G.A. Kluge. ZI 32, Kara Sea, 77°16'N, 94°56'E, 22.09.1933, Sibiriakov, Stn 38, collected by G.I. Gorbunov, determined by G.A. Kluge. ZI 33, Kara Sea, 74°12'N, 59°54'E, 1934, Sedov, Stn 99, collected by V.L. Vagin, determined by G.A. Kluge. ZI 54/888, Polar Basin, SP-3, Stn 87, 1948, collected by A.A.R.I., determined by M.G. Petrovskaya. ZI 55/2534, Laptev Sea, 77°10.3'N, 120°50'E, depth 220 m, 19.08.1937, Sadko, Stn 14, collected by G.I. Gorbunov, determined by G.A. Kluge. ZI 56/2578, Greenland Sea, 80°1.5'N, 9°17'W, depth 500 m, 1.08.1935, Sadko, Stn 11\34, collected by G.I. Gorbunov, determined by G.A. Kluge. ZI 57/2650, Greenland Sea, 80°30.6'N, 9°59'W, depth 680 m, 3.08.1935, Sadko, Stn 13\38, collected by G.I. Gorbunov, determined by G.A. Kluge. ZI 58/2696, Polar Basin between FJL and Spitsbergen, 80°45.6'N, 29°57'E, depth 445 m, 2.08.1935, Sadko, Stn 18\50, collected by G.I. Gorbunov, determined by G.A. Kluge. ZI 59/3058, Polar Basin, 81°1'N, 19°28'E, depth 180 m, 18.08.1899, Ermak, Stn 28, collected by A.G. Chernyshov, determined by G.A. Kluge. ZI 60/5426, Polar Basin, 82°20'N, 47°17'E, 14.09.1955, Litke, Stn 9, collected by V.M. Koltun, determined by M.G. Gostilovskaya. 61/5427, Polar Basin, 82°22'N, 54°14'E, 14.09.1955, Litke, Stn 11, collected by V.M. Koltun, determined by M.G. Gostilovskaya. 62/5520, Polar Basin, 80°47'N, 47°17.41'E, 10.10.1955, Litke, Stn 47, collected by V.M. Koltun, determined by M.G. Gostilovskaya. 63/5521, Polar Basin, 80°58'N, 17°17'E, 10.10.1955, Litke, Stn 46, collected by V.M. Koltun, determined by M.G. Gostilovskaya. ZI 64/50539, 64º45.2776'N, 05º5.6080'E, depth 718 m, 22.05.2006, Pourquoi Pas, Stn 271-01, Pruer-2, collected in frame of HERMES program, cruise leader H. Nouze, determined by N.V. Denisenko. ZI 65/50540, 64º44.5438'N, 04º47.9748'E, depth 798 m, 23.05.2006, Pourquoi Pas, Stn 271-01, ASPI-7, collected in frame of HERMES program by H. Nouze, determined by N.V. Denisenko.

**Unregistered material**

### Faroe Islands, 61°13.30’N, 4°46.50’W, depth 780 m, 19.07.1987, Stn 41, BIOFAR project, Rothlisberg & Pearcy epibenthic sampler, determined P.J. Hayward. Faroe Islands, 62°19.12’N, 3°54.79’W, depth 507 m, 09.05.1988, Stn 172, BIOFAR project, Rothlisberg & Pearcy epibenthic sampler, determined P.J. Hayward. Faroe Islands, 63°00.79’N, 7°49.22’W, depth 698 m, 16.05.1988, Stn 274, BIOFAR project, detritus sledge, determined P.J. Hayward. Faroe Islands, 62°59.42’N, 6°57.52’W, depth 910 m, 04.06.1989, Stn 459, BIOFAR project, detritus sledge, determined P.J. Hayward. Faroe Islands, 61°03.66’N, 4°43.92’W, depth 973 m, 21.07.1989, Stn 478, BIOFAR project, detritus sledge, determined P.J. Hayward. Faroe Islands, 61°00.16’N, 5°13.89’W, depth 604 m, 22.06.1989, Stn 481, BIOFAR project, detritus sledge, determined P.J. Hayward. Faroe Islands, 61°09.90’N, 5°01.80’W, depth 610 m, 28.09.1990, Stn 719, BIOFAR project, detritus sledge, determined P.J. Hayward. Faroe Islands, 61°07.00’N, 4°58.30’W, depth 700 m, 28.09.1990, Stn 720, BIOFAR project, detritus sledge, determined P.J. Hayward. Faroe Islands, 61°07.20’N, 4°40.60’W, depth 1014 m, 28.09.1990, Stn 723, BIOFAR project, detritus sledge, determined P.J. Hayward. Faroe Islands, 60°32.20’N, 7°08.20’W, depth 949 m, 29.09.1990, Stn 730, BIOFAR project, detritus sledge, determined P.J. Hayward. Faroe Islands, 62°46.80’N, 5°51.50’W, depth 497 m, 03.10.1990, Stn 749, BIOFAR project, detritus sledge, determined P.J. Hayward. Faroe Islands, 62°48.70’N, 5°44.10’W, depth 600 m, 03.10.1990, Stn 750, BIOFAR project, detritus sledge, determined P.J. Hayward. Faroe Islands, 62°49.80’N, 5°35.00’W, depth 708 m, 03.10.1990, Stn 751, BIOFAR project, detritus sledge, determined P.J. Hayward. Faroe Islands, 62°50.00’N, 5°28.00’W, depth 809 m, 03.10.1990, Stn 752, BIOFAR project, detritus sledge, determined P.J. Hayward. Faroe Islands, 62°19.90’N, 3°06.00’W, depth 645 m, 06.10.1990, Stn 769, BIOFAR project, detritus sledge, determined P.J. Hayward. Faroe Islands, 62°24.70’N, 3°52.20’W, depth 560 m, 08.10.1990, Stn 774, BIOFAR project, detritus sledge, determined P.J. Hayward.

***Pseudoflustra birulai***

**Registered material**

ZI 1, Kara Sea, 80°26’N, 64°14’E, 2.08.1901, *Ermak*, determined by G.A. Kluge, collected by A.G. Chernyshov. ZI 2, Taymyr Bay, 76°59,5'N, 100°19,5'E, Stn 44, 18.08.1901, *Zaria*, R.P.E., determined by G.A. Kluge. ZI 3, Kara Sea, 76°00' N, 91°12'E, 28.08.1933, *Sibiriakov*, Stn 23, collected by G.P. Gorbunov, determined by G.A. Kluge. ZI 4, Kara Sea, 77°46'N, 103°48'E, 30.08.1933, *Sibiriakov*, Stn 27, collected by G.P. Gorbunov, determined by G.A. Kluge. ZI 5, Kara Sea, 70°50'N, 57°34'E, 13.08.1931, *Rusanov*, Stn 17, collected by G.P. Gorbunov, determined by G.A. Kluge. ZI 6, 78°49'N, 100°10'E, Shokalskiy Strait, Severnaya Zemlia, depth 276 m, 19.08.1932, *Rusanov*, Stn 6, collected by N.N. Kondakov, V.L. Vagin, determined by G.A. Kluge. ZI 7, Kara Sea, 73°39’N, 62°37’E, *Rusanov*, trawl, 19.09.1931, collected by G.P. Gorbunov, determined by G.A. Kluge. ZI 8, 77°49,5'N, 102°16'E, Wilkitskiy Strait, 7.09.1932, *Rusanov*, Stn 29, collected by N.N. Kondakov, V.L. Vagin, determined by G.A. Kluge. ZI 9, Kara Sea, 73°39’N, 62°37’E, *Rusanov*, trawl, 19.09.1931, collected by G.P. Gorbunov, determined by G.A. Kluge. ZI 10 and ZI 11Kara Sea, 79°55'N, 88°58'E, 31.08.1930, *Sedov*, Stn 57, collected by G.P. Gorbunov, determined by G.A. Kluge. ZI 12, Kara Sea, 80°26'N, 88°57'E, 31.08.1930, *Sedov*, Stn 58, collected by G.P. Gorbunov, determined by G.A. Kluge. ZI 13, Kara Sea, 78°33'N, 63°10'E, 5.09.1929, *Sedov*, Stn 25, collected by G.P. Gorbunov, determined by G.A. Kluge. ZI 14, Wilkitzki Strait, 77°56’N, 103°33’E, Stn 26, *Rusanov*, trawl, 6-7.09.1932, collected by V.L. Vagin determined by G.A. Kluge. ZI 15, Kara Sea, 77°16'N, 94°568'E, 22.09.1933, *Sibiriakov*, Stn 38, collected by G.P. Gorbunov, determined by G.A. Kluge. ZI 16, Kara Sea, 74°12'N, 59°54'E, 1934, *Sedov*, Stn 99, collected by V.L. Vagin, determined by G.A. Kluge. ZI 17/5430, Polar Basin, 82°20'N, 47°17'E, 14.09.1955, *Litke*, Stn 9, collected by V.M. Koltun, determined by G.A. Kluge. ZI 28/1739, Polar Basin, 79°30'N, 150°10'E, 12.04.1938, *Sadko*, Stn 93, collected by G.P. Gorbunov, determined by G.A. Kluge. ZI 29/2577, Kara Sea, 81°33.1'N, 75°57'E, 1.09.1935, *Sadko*, Stn 33/77, collected by G.P. Gorbunov, determined by G.A. Kluge. ZI 30/2579, Polar Basin, 80°09.9'N, 33°59.5'E, 16.08.1935, *Sadko*, Stn 24/55, collected by G.P. Gorbunov, determined by G.A. Kluge. ZI 31/2585, Kara Sea, 79°01.5'N, 75°08'E, 26.08.1935, *Sadko*, Stn 35/65, collected by G.P. Gorbunov, determined by G.A. Kluge. ZI 32/2588, 70°50'N, 57°34'E, near Kara Gate Strait, depth 93 m, 13.08.1931, *Rusanov*, Stn 17, collected by G.P. Gorbunov, determined by G.A. Kluge. ZI 33/5431, Polar Basin, 82°11'N, 60°57'E, 15.09.1955, *Litke*, collected by V.M. Koltun, determined by M.G. Gostilovskaya. ZI 34/4899, Polar Basin, 82°24'N, 58°25'E, 14.09.1955, *Litke*, Stn 12, collected by V.M. Koltun, determined by M.G. Gostilovskaya. ZI 35/6212, Polar Basin, 82°00'N, 42°00'E, 18.09.1955, *Litke*, Stn 28, collected by V.M. Koltun, determined by M.G. Gostilovskaya. ZI 36/13836, Greenland Sea, 77°51'N, 05°59'W, 25.03.1958, *Lena*, collected by M. Petrovskaya, determined by M.G. Gostilovskaya. NHM 2012.3.7.1, Belgica Bank, East Greenland, 79°19.58’N, 12°13.79’W, depth 168 m, 12.08.2000, Stn 239, *Polarstern*, cruise ANTXVI/2, Agassiz trawl, collected by B. Bader.

***Pseudoflustra minima***

**Unregistered material**

IMB (Bergen), Norwegian Sea, MARIANO program, 11.06.2008 St. R228-09, 69º5.39’N, 13º41.58’E, Beam trawl, depth 640 m, det. N.V. Denisenko. IMB (Bergen), Norwegian Sea, MARIANO program, 11.06.2008, St. R228-12, 69º06’N, 13º44,4’E, RP-sledge, depth 661.9 m, det. N.V. Denisenko. IMB (Bergen), Norwegian Sea, MARIANO program, St. R296-34. 13.10.2008, 68º37.074’N, 12º41.557’E, RP-sledge, depth 197 m, det. N.V. Denisenko.

***Pseudoflustra virgula***

**Unregistered material**

Faroe Islands, 60°58.51’N, 9°55.84’W, depth 1083 m, 23.07.1989, Stn 490, BIOFAR project, detritus sledge, det. P.J. Hayward. Faroe Islands, 60°40.05’N, 11°40.56’W, depth 914 m, 26.07.1989, Stn 516, BIOFAR project, detritus sledge, det. P.J. Hayward.
